# A New Application of Parallel Synthesis Strategy for Discovery of Amide-Linked Small Molecules as Potent Chondroprotective Agents in TNF-α-Stimulated Chondrocytes

**DOI:** 10.1371/journal.pone.0149317

**Published:** 2016-03-10

**Authors:** Chia-Chung Lee, Yang Lo, Ling-Jun Ho, Jenn-Haung Lai, Shiu-Bii Lien, Leou-Chyr Lin, Chun-Liang Chen, Tsung-Chih Chen, Feng-Cheng Liu, Hsu-Shan Huang

**Affiliations:** 1 Graduate Institute of Cancer Biology and Drug Discovery, College of Medical Science and Technology, Taipei Medical University, Taipei, Taiwan; 2 School of Pharmacy, National Defense Medical Center, Taipei, Taiwan; 3 Graduate Institute of Medical Science, National Defense Medical Center, Taipei, Taiwan; 4 Institute of Cellular and System Medicine, National Health Research Institute, Zhunan, Taiwan; 5 Division of Allergy, Immunology and Rheumatology, Department of Internal Medicine, Chang Gung Memorial Hospital, Chang Gung University, Tao-Yuan, Taiwan; 6 Department of Orthopaedics, Tri-Service General Hospital, National Defense Medical Center, Taipei, Taiwan; 7 Rheumatology/Immunology/Allergy, Tri-Service General Hospital, National Defense Medical Center, Taipei, Taiwan; University of Umea, SWEDEN

## Abstract

As part of an effort to profile potential therapeutics for the treatment of inflammation-related diseases, a diversity of amide-linked small molecules was synthesized by using parallel synthesis strategy. Moreover, these new compounds were also evaluated for their inhibitory effects on nitric oxide (NO) by using tumor necrosis factor alpha (TNF-α)-induced inflammatory responses in chondrocytes. Among the tested compounds, *N*-(3-chloro-4-fluorophenyl)-2-hydroxybenzamide (HS-Ck) was the most potent inhibitor of NO production and inducible nitric oxide synthase (iNOS) expression in TNF-α-stimulated chondrocytes. In addition, our biological results indicated that HS-Ck might suppress the expression levels of iNOS and matrix metalloproteinases-13 (MMP-13) activities through downregulating the activation of nuclear factor kappa B (NF-κB) and signal transducer and activator of transcription 3 (STAT-3) transcriptional factors. Therefore, the parallel synthesis was successful used to develop a new class of potential anti-inflammatory agents as chondroprotective candidates for the treatment of osteoarthritis.

## Introduction

Osteoarthritis (OA) is characterized by breakdown of collagen and aggrecan in the cartilage, which leads to chronic joint pain and disability in the middle aged and elderly patients [1, 2]. Many catabolic factors such as pro-inflammatory cytokines, matrix metalloproteinases (MMPs), and nitric oxide (NO) can lead to the progressive destruction of joints in OA development [3–6]. The tumor necrosis factor alpha (TNF-α) is the one of the most important catabolic cytokine in the pathogenesis of OA [7–9]. Through the activation of TNF-α–induced signaling transduction cascades, it can activate various transcriptional factors, including the nuclear factor kappa B (NF-κB) [10, 11] and the signal transducer and activator of transcription 3 (STAT-3) [12, 13], which paly critical roles in the inflammation-mediated diseases. In addition, the accumulating studies report that the TNF-α is a vital cell signaling cytokine in the progression of cartilage degradation releasing several inflammatory cytokines, inflammatory mediator NO, and MMPs such as MMP-3 (stromelysin-1) and MMP-13 (collagenase-3) [14–16]. Furthermore, the degradation of type II collagen by enzymatic cleavages (MMPs) is an essential step in the loss of integrity of cartilage, especially for collagenase-3 (MMP-13) [17]. Hence, the modulation of these catabolic factors provides a principal target for the treatment of OA diseases in the development of anti-inflammatory drugs.

**Fig 1:**
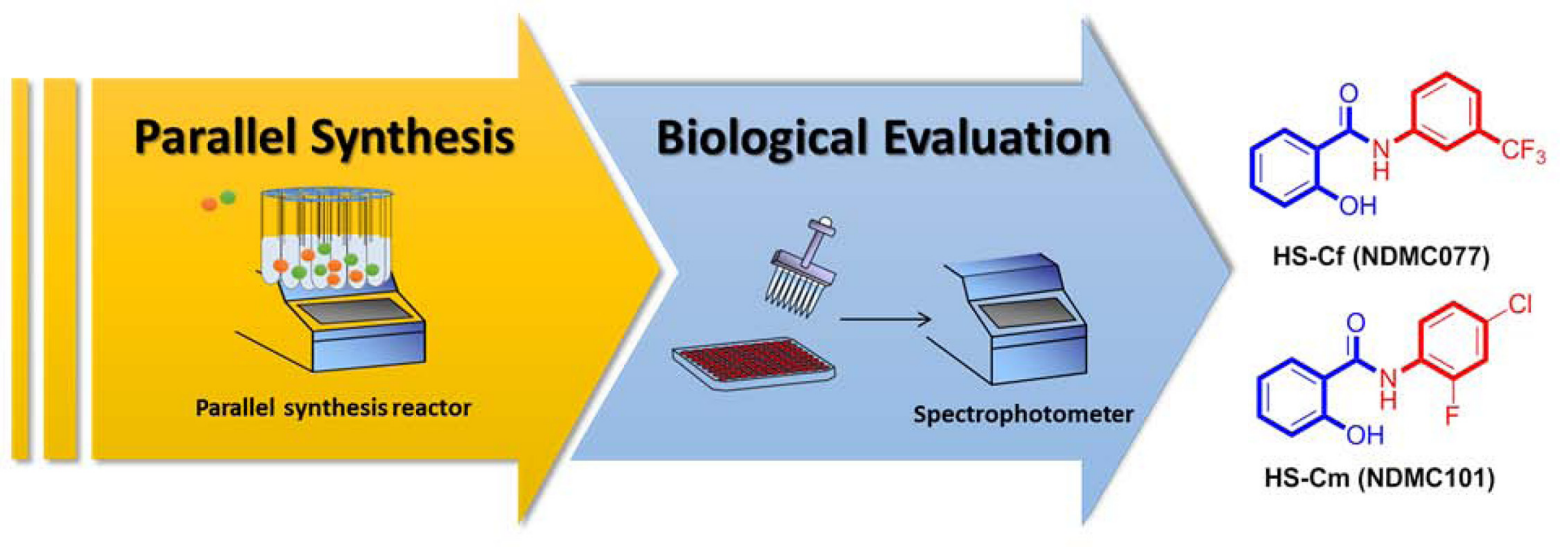
Two potent amide-linked small molecules (HS-Cf and HS-Cm) were identified their biological activities in our previous studies.

The “combinatorial chemistry” concept has been adopted as an efficient technique to synthesize, in parallel synthesis, more than one compound [18–20]. In addition, the parallel synthesis approach has been widely used as a powerful method in the drug development [21, 22]. Taking advantage of the concept of combinatorial chemistry, our previous studies have identified two potent amide-linked small molecules (HS-Cf and HS-Cm) from a mini-library chemical bank (containing more than 300 small molecules) by using TNF-α-induced inflammatory responses in chondrocytes as a screening tool (Fig 1) [23–25]. In our previous study, HS-Cf (NDMC077) has been found to have the chondroprotective effect that could prevent TNF-α-induced cartilage destruction through downregulating the interferon regulatory factor-1 (IRF-1) signaling [25]. Moreover, our recent reports showed that HS-Cm (NDMC101) has been found to be a potent immunomodulatory agent with anti-inflammatory activities in inflammation-related diseases [23, 24]. All encourage results and previous substantial efforts initiated quest for the development of anti-inflammatory agents, which can prevent TNF-α-induced cartilage damage in OA diseases.

**Fig 2:**
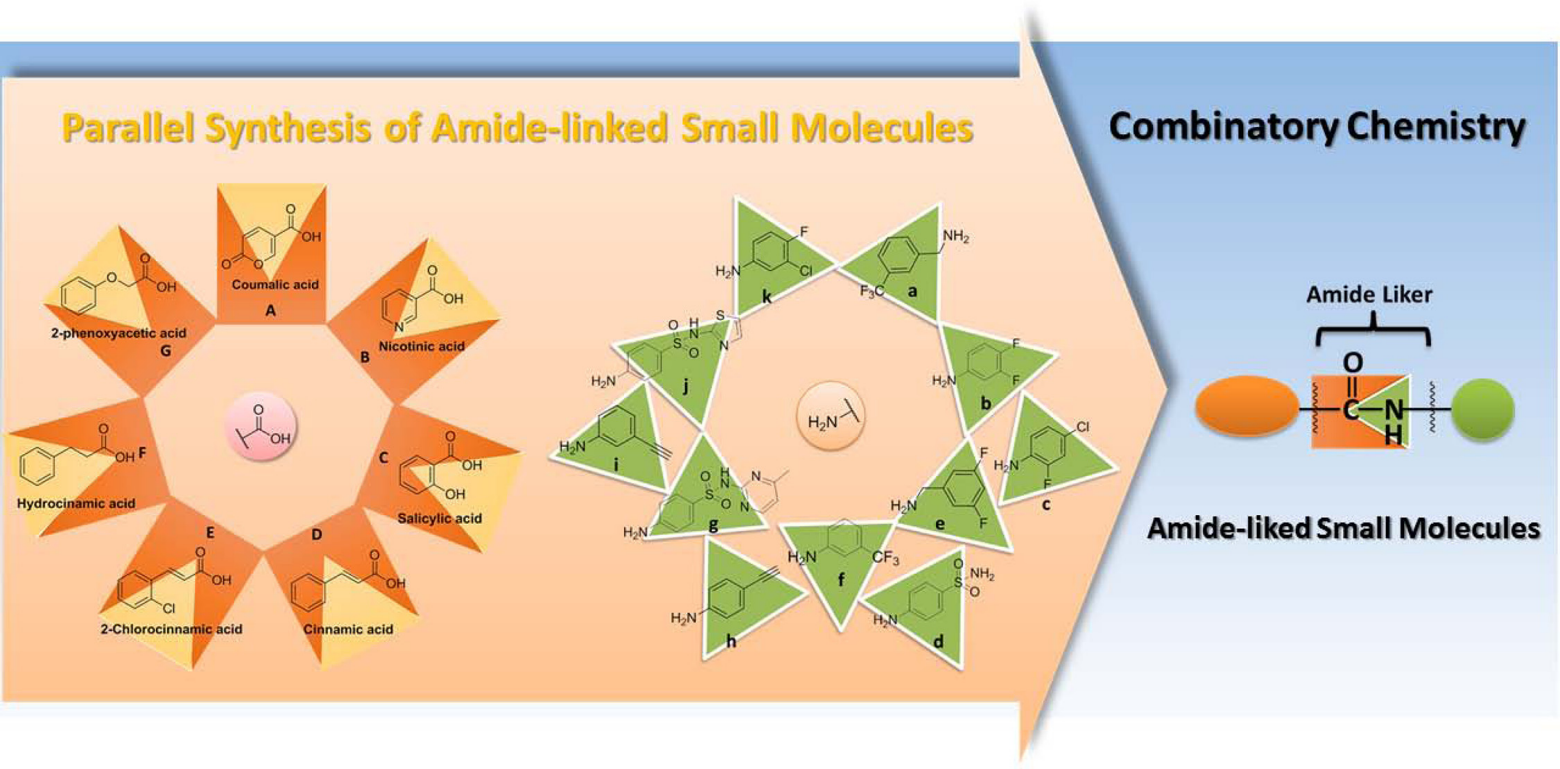
Synthesis of amide-linked small molecules by using parallel synthesis approach.

In the present work, the parallel synthesis approach was used to prepare a diverse range of amide-linked small molecules through coupling different core structures of carboxylic acids with appropriate amines (Fig 2). These new compounds were also evaluated their anti-inflammatory effects on TNF-α-induced NO production in chondrocytes. On the basis of primary screening results, HS-Ck was found to be a potent chondroprotective agent through suppressing TNF-α-induced NO production and iNOS expression in chondrocytes. In addition, the chondroprotective effects of HS-Ck on the activation of TNF-αinduced signaling transduction were further identified by reducing the MMP-13 expression and decreasing the transcriptional activation of NF-κB and STAT-3. In this study, HS-Ck can be considered as a potent structure for the development of chondroprotective agents to disrupt the TNF-α-mediated inflammatory responses in the pathogenesis of OA diseases.

## Materials and Methods

### Chemistry

Unless otherwise stated, all materials used were commercially available. Chemical reagents and solvents were purchased from ALDRICH and MERCK without further purification. Reactions requiring anhydrous conditions were performed in oven-dried glassware and cooled under nitrogen atmosphere. The parallel synthesis reactor was used using the BÜCHI SynCore^®^ reactor. Melting points were determined by the BÜCHI B-545 melting point apparatus and are uncorrected. Analytical thin layer chromatography was performed with E. MERCK silica gel 60 F_254_. ^1^H Nuclear magnetic resonance (NMR) and ^13^C NMR spectra were recorded on AGILENT 400 MR DD2 (400 MHz). *δ* value is presented in parts per million (ppm) relative to TMS as an internal standard (0 ppm). Coupling constants (*J*) are expressed in Hz. Multiplicities were recorded as singlet (s), doublet (d), triplet (t), and double of doublet (dd). High resolution mass spectra (HRMS) were measured by FINNIGAN MAT-95XL (high resolution electron impact ionization, HREI) and FINNIGAN MAT-95S (high resolution electrospray ionization, HRESI). Spectral data are recorded as *m/z* values.

### General Procedure for the Preparation of Compounds

To a solution of carboxylic acids (2 mmol) in methylene chloride (10 mL), solid 1-hydroxybenzotriazole monohydrate (0.27 g, 2 mmol) and *N*-ethyl-*N*’-(3-dimethylaminopropyl) carbodiimide hydrochloride (0.38 g, 2 mmol) were added. The mixture solutions were reacted with various anilines (4 mmol) and then stirred at room temperature for 3 days in parallel synthesis reactor. The reaction mixture was evaporated to dryness under reduced pressure and the residue was extraction with ethyl acetate, washed with 10% NaHCO_3_, and H_2_O. The organic phase was separated and dried with anhydrous MgSO_4_, and dried *in vacuo*. The crude product was washed and purified by crystallization from hot ethanol and methylene chloride to obtain title compounds.

#### 2-Oxo-*N*-(3-(trifluoromethyl)benzyl)-2*H*-pyran-5-carboxamide (Aa)

The pure compound was obtained as orange powder (yield 38%). Mp: 159–160°C (EtOH); ^1^H NMR (400 MHz, DMSO-*d*_*6*_): *δ* ppm 4.69 (d, *J* = 6.4 Hz, 2H), 7.47 (d, *J* = 9.2 Hz, 1H), 7.60–7.70 (m, 4H), 7.75 (s, 1H), 8.17 (d, *J* = 14.4 Hz, 1H), 10.10 (t, *J* = 6.8 Hz, 1H); ^13^C NMR (100 MHz, DMSO-*d*_*6*_): *δ* ppm 93.30, 101.28, 101.46, 103.29, 104.35, 110.95, 111.21, 122.80, 128.47, 129.37, 130.80, 133.25, 139.74, 148.52, 149.02, 149.16, 152.83, 160.42, 160.70, 161.52, 163.67; HRMS (ESI) *m/z*:calcd [M]^+^, 297.0613 (C_14_H_10_F_3_NO_3_^+^), found [M-H]^+^, 296.0550 (C_14_H_9_F_3_NO_3_^+^).

#### *N*-(4-Ethynylphenyl)-2-oxo-2*H*-pyran-5-carboxamide (Ah)

The pure compound was obtained as orange powder (yield 41%). Mp: 157–158°C (EtOH); ^1^H NMR (400 MHz, DMSO-*d*_*6*_): *δ* ppm 4.23 (s, 1H), 7.51–7.55 (m, 5H), 7.63 (d, *J* = 9.2 Hz, 1H), 8.58 (d, *J* = 14 Hz, 1H), 11.13 (d, *J* = 13.6 Hz, 1H); ^13^C NMR (100 MHz, DMSO-*d*_*6*_): *δ* ppm 81.46, 83.01, 96.11, 104.15, 118.76, 130.03, 133.10, 138.99, 148.485, 152.279, 161.514, 163.38; HRMS (ESI) *m/z*:calcd [M]^+^, 239.0582 (C_14_H_9_NO_3_^+^), found [M+H]^+^, 240.0655 (C_14_H_10_NO_3_^+^).

#### *N*-(4-Chloro-2-fluorophenyl)nicotinamide (Bc)

The pure compound was obtained as white powder (yield 39%). Mp: 180–182°C (EtOH); ^1^H NMR (400 MHz, DMSO-*d*_*6*_): *δ* ppm 7.33 (d, *J* = 8.8 Hz, 1H), 7.54–7.58 (m, 2H), 7.67 (t, *J* = 8.4 Hz, 1H), 8.29 (d, *J* = 8.2 Hz, 1H), 8.77 (dd, *J* = 6.4, 1.2 Hz, 1H), 9.10 (d, *J* = 2 Hz, 1H), 10.42 (s, 1H); ^13^C NMR (100 MHz, DMSO-*d*_*6*_): *δ* ppm 116.55, 116.79, 123.75, 124.63, 124.77, 128.07, 129.51, 130.42, 1135.76, 148.87, 152.60, 154.32, 156.82, 164.34; HRMS (ESI) *m/z*:calcd [M]^+^, 250.0309 (C_12_H_8_ClFN_2_O^+^), found [M-H]^+^, 249.0236 (C_12_H_7_ClFN_2_O^+^).

#### *N*-(4-Sulfamoylphenyl)nicotinamide (Bd)

The pure compound was obtained as white powder (yield 31%). Mp: 238–241°C (EtOH); ^1^H NMR (400 MHz, DMSO-*d*_*6*_): *δ* ppm 7.92 (s, 2H), 7.57–7.60 (m, 1H), 7.81 (d, *J* = 8.8 Hz, 2H), 7.94 (d, *J* = 8.8 Hz, 2H), 8.30 (td, *J* = 8, 2 Hz, 1H), 8.77 (dd, *J* = 4.8, 1.2 Hz, 1H), 9.11 (d, *J* = 1.6 Hz, 1H), 10.73 (s, 1H); ^13^C NMR (100 MHz, DMSO-*d*_*6*_): *δ* ppm 120.06, 123.71, 126.69, 130.31, 135.75, 139.13, 141.86, 148.82, 152.49, 164.65; HRMS (ESI) *m/z*:calcd [M]^+^, 277.0521 (C_12_H_11_N_3_O_3_S^+^), found [M-H]^+^, 276.0448 (C_12_H_10_N_3_O_3_S^+^).

#### *N*-(3-(Trifluoromethyl)phenyl)nicotinamide (Bf)

The pure compound was obtained as orange powder (yield 43%). Mp: 178–179°C (EtOH); ^1^H NMR (400 MHz, DMSO-*d*_*6*_): *δ* ppm 7.48 (d, *J* = 8 Hz, 1H), 7.57–7.63 (m, 2H), 8.03 (d, *J* = 8.4 Hz, 1H), 8.24 (s, 1H), 8.29–8.32 (m, 1H), 8.78 (d, *J* = 3.6 Hz, 1H), 9.12 (d, *J* = 1.2 Hz, 1H), 10.73 (s, 1H); ^13^C NMR (100 MHz, DMSO-*d*_*6*_): *δ* ppm 116.37, 116.42, 120.35, 122.80, 123.63, 123.86, 129.28, 129.59, 130.05, 130.19, 135.59, 139.66, 148.76, 152.45, 164.55; HRMS (ESI) *m/z*:calcd [M]^+^, 266.0667 (C_13_H_9_F_3_N_2_O^+^), found [M+H]^+^, 267.0740 (C_13_H_10_F_3_N_2_O ^+^).

#### *N*-(3,4-Difluorophenyl)-2-hydroxybenzamide (Cb)

The pure compound was obtained as white powder (yield 36%). Mp: 191–192°C (EtOH); ^1^H NMR (400 MHz, DMSO-*d*_*6*_): *δ* ppm 6.20–6.98 (m, 2H), 7.39–7.49 (m, 3H), 7.86–7.93 (m, 2H), 10.61 (s, 1H); ^13^C NMR (100 MHz, DMSO-*d*_*6*_): *δ* ppm 109.78, 109.99, 117.18, 117.32, 117.37, 117.55, 117.91, 119.01, 129.24, 133.76, 146.97, 147.09, 147.81, 147.68, 158.33, 166.55; HRMS (ESI) *m/z*:calcd [M]^+^, 239.0582 (C_13_H_9_F_2_NO_2_^+^), found [M-H]^+^, 248.0527 (C_13_H_8_F_2_NO_2_^+^).

#### *N*-(3-Ethynylphenyl)-2-hydroxybenzamide (Ci)

The pure compound was obtained as white powder (yield 24%). Mp: 175–176°C (EtOH); ^1^H NMR (400 MHz, DMSO-*d*_*6*_): *δ* ppm 6.92–6.98 (m, 2H), 7.22 (d, *J* = 7.6 Hz, 1H), 7.36 (t, *J* = 8 Hz, 1H), 7.42 (td, *J* = 7.6, 1.6 Hz, 1H), 7.69 (dd, *J* = 8.2, 1.2 Hz, 1H), 7.88 (t, *J* = 1.6 Hz, 1H), 7.90 (dd, *J* = 8, 1.6 Hz, 1H), 10.42 (s, 1H), 11.62 (s, 1H); ^13^C NMR (100 MHz, DMSO-*d*_*6*_): *δ* ppm 80.83, 83.39, 117.30, 11785, 119.27, 121.58, 122.15, 123.76, 127.47, 129.26, 129.37, 133.86, 138.56, 158.26, 166.75; HRMS (ESI) *m/z*:calcd [M]^+^, 237.0790 (C_15_H_11_NO_2_^+^), found [M-H]^+^, 236.0722 (C_15_H_10_NO_2_^+^).

#### *N*-(3-Chloro-4-fluorophenyl)-2-hydroxybenzamide (Ck)

The pure compound was obtained as white powder (yield 42%). Mp: 203–204°C (EtOH); ^1^H NMR (400 MHz, DMSO-*d*_*6*_): *δ* ppm 6.94–6.99 (m, 2H), 7.40–7.45 (m, 2H), 7.62–7.66 (m, 1H), 7.88 (dd, *J* = 7.6, 1.2 Hz, 1H), 8.03 (dd, *J* = 6.8, 2.8 Hz, 1H); 11.53 (s, 1H); ^13^C NMR (100 MHz, DMSO-*d*_*6*_): *δ* ppm 116.81, 117.21, 117.77, 119.17, 119.25, 121.23, 121.30, 122.38, 129.14, 133.78, 135.50, 135.54, 154.88, 158.10, 166.60; HRMS (ESI) *m/z*:calcd [M]^+^, 265.0306 (C_13_H_9_ClFNO_2_^+^), found [M+H]^+^, 266.0379 (C_13_H_10_ClFNO_2_^+^).

#### *N*-(3-(Trifluoromethyl)benzyl)cinnamamide (Da)

The pure compound was obtained as white powder (yield 67%). Mp: 87–88°C (EtOH); ^1^H NMR (400 MHz, DMSO-*d*_*6*_): *δ* ppm 4.49 (d, *J* = 6 Hz, 2H), 6.53 (d, *J* = 16 Hz, 1H), 6.70 (d, *J* = 16 Hz, 1H), 7.36–7.43 (m, 3H), 7.49 (d, *J* = 16 Hz, 1H), 8.74 (t, *J* = 6 Hz, 1H), 12.41 (s, 1H); ^13^C NMR (100 MHz, DMSO-*d*_*6*_): *δ* ppm 41.90, 119.23, 121.76, 123.62, 123.65, 123.81, 123.85, 127.64, 128.23, 128.97, 129.47, 129.60, 130.25, 131.55, 134.25, 134.80, 139.33, 141.03, 143.98, 165.21, 167.63; HRMS (EI) *m/z*:calcd [M]^+^, 305.1027 (C_17_H_14_F_3_NO^+^), found [M]^+^, 305.1027 (C_17_H_14_F_3_NO^+^).

#### *N*-(3,4-Difluorophenyl)cinnamamide (Db)

The pure compound was obtained as white powder (yield 75%). Mp: 127–128°C (EtOH); ^1^H NMR (400 MHz, DMSO-*d*_*6*_): *δ* ppm 6.77 (d, *J* = 16 Hz, 1H), 7.37–7.47 (m, 3H), 7.51–7.55 (m, 1H), 7.59–7.64 (m, 2H), 7.71 (d, *J* = 8.4 Hz, 1H), 7.97 (d, *J* = 8 Hz, 1H), 10.44 (s, 1H); ^13^C NMR (100 MHz, DMSO-*d*_*6*_): *δ* ppm 108.13, 108.34, 109.65, 115.56, 117.52, 117.70, 119.23, 121.65, 124.59, 127.42, 127.88, 129.10, 130.04, 134.54, 136.27, 136.39, 140.88, 163.78; HRMS (ESI) *m/z*:calcd [M]^+^, 259.0809 (C_15_H_11_F_2_NO ^+^), found [M-H]^+^, 258.0738 (C_15_H_10_F_2_NO ^+^).

#### *N*-(2-Chloro-4-fluorophenyl)cinnamamide (Dc)

The pure compound was obtained as white crystal (yield 47%). Mp: 161–162°C (EtOH); ^1^H NMR (400 MHz, DMSO-*d*_*6*_): *δ* ppm 7.06 (d, *J* = 15.6 Hz, 1H), 7.27–7.30 (m, 1H), 7.39–7.47 (m, 3H), 7.51 (dd, *J* = 10.8, 2.4 Hz, 1H), 7.58–7.63 (m, 3H), 8.16 (t, *J* = 8.8 Hz, 1H), 10.05 (s, 1H); ^13^C NMR (100 MHz, DMSO-*d*_*6*_): *δ* ppm 116.05, 116.24, 121.55, 124.35, 124.59, 124.63, 125.63, 1125.74, 127.85, 129.06, 129.99, 134.62, 141.05, 151.81, 164.02; HRMS (EI) *m/z*:calcd [M]^+^, 275.0513 (C_15_H_11_ClFNO^+^), found [M]^+^, 275.0519 (C_15_H_11_ClFNO^+^).

#### *N*-(4-Sulfamoylphenyl)cinnamamide (Dd)

The pure compound was obtained as white powder (yield 38%). Mp: 270–271°C (EtOH); ^1^H NMR (400 MHz, DMSO-*d*_*6*_): *δ* ppm 6.84 (d, *J* = 15.6 Hz, 1H), 7.27 (s, 2H), 7.41–7.47 (m, 3H), 7.61–7.65 (m, 3H), 7.78–7.87 (m, 4H), 10.55 (s, 1H); ^13^C NMR (100 MHz, DMSO-*d*_*6*_): *δ* ppm 118.83, 121.78, 126.82, 127.89, 129.10, 130.06, 134.55, 138.43, 141.08, 142.18, 164.01; HRMS (EI) *m/z*:calcd [M]^+^, 302.0725 (C_15_H_14_N_2_O_3_S^+^), found [M]^+^, 302.0732 (C_15_H_14_N_2_O_3_S^+^).

#### *N*-(3,5-Difluorobenzyl)cinnamamide (De)

The pure compound was obtained as white powder (yield 52%). Mp: 122–123°C (EtOH); ^1^H NMR (400 MHz, DMSO-*d*_*6*_): *δ* ppm 4.41 (d, *J* = 6 Hz, 2H), 6.69 (d, *J* = 18.8 Hz, 1H), 6.98–7.02 (m, 2H), 7.10 (tt, *J* = 9.2, 2.4 Hz, 1H), 7.35–7.44 (m, 3H), 7.48 (d, *J* = 16 Hz, 1H), 7.48 (d, *J* = 16 Hz, 1H), 7.58 (dd, *J* = 7.6, 1.2 Hz, 2H), 8.70 (t, *J* = 5.6 Hz, 1H); ^13^C NMR (100 MHz, DMSO-*d*_*6*_): *δ* ppm 41.65, 102.01, 102.27, 102.52, 110.11, 110.18, 110.30, 110.36, 121.69, 127.66, 128.99, 129.64, 134.79, 139.41, 141.46, 144.29, 144.38, 161.12, 161.25, 163.57, 163.70, 165.27; HRMS (ESI) *m/z*:calcd [M]^+^, 273.0965 (C_16_H_13_F_2_NO^+^), found [M-H]^+^, 272.0892 (C_16_H_12_F_2_NO^+^).

#### *N*-(3-(Trifluoromethyl)phenyl)cinnamamide (Df)

The pure compound was obtained as orange powder (yield 82%). Mp: 86–87°C (EtOH); ^1^H NMR (400 MHz, DMSO-*d*_*6*_): *δ* ppm 6.83 (d, *J* = 15.6 Hz, 1H), 7.40–7.47 (m, 4H), 7.55–7.65 (m, 4H), 7.87 (d, *J* = 8.4 Hz, 1H), 8.22 (s, 1H), 10.58 (s, 1H); ^13^C NMR (100 MHz, DMSO-*d*_*6*_): *δ* ppm 115.24, 115.28, 119.66, 119.69, 121.71, 122.76, 127.87, 128.22, 129.07, 130.02, 130.08, 134.52, 140.04, 140.97143.96, 164.05, 167.61; HRMS (ESI) *m/z*:calcd [M]^+^, 291.0871 (C_16_H_12_F_3_NO^+^), found [M]^+^, 291.0874 (C_16_H_12_F_2_NO^+^).

#### *N*-(3-Ethynylphenyl)cinnamamide (Di)

The pure compound was obtained as white powder (yield 89%). Mp: 133–134°C (EtOH); ^1^H NMR (400 MHz, DMSO-*d*_*6*_): *δ* ppm 4.19 (s, 1H), 6.81 (d, *J* = 16 Hz, 1H), 7.33–7.47 (m, 5H), 7.62–7.66 (m, 3H), 7.71 (d, *J* = 8.4 Hz, 1H), 7.97 (d, *J* = 8.4 Hz, 1H), 10.32 (s, 1H); ^13^C NMR (100 MHz, DMSO-*d*_*6*_): *δ* ppm 80.71, 83.44, 119.85, 121.95, 122.02, 122.11, 124.55, 126.65, 127.42, 127.84, 129.09, 129.36, 129.96, 134.61, 139.50, 140.65, 163.79; HRMS (ESI) *m/z*:calcd [M]^+^, 247.0997 (C_17_H_13_NO^+^), found [M-H]^+^, 246.0924 (C_17_H_12_NO^+^).

#### *N*-(4-(*N*-(Thiazol-2-yl)sulfamoyl)phenyl)cinnamamide (Dj)

The pure compound was obtained as white powder (yield 57%). Mp: 286–287°C (EtOH); ^1^H NMR (400 MHz, DMSO-*d*_*6*_): *δ* ppm 6.81 (d, *J* = 4.4 Hz, 1H), 6.83 (d, *J* = 15.6 Hz, 1H), 7.24 (d, *J* = 4.8 Hz, 1H), 7.41–7.47 (m, 3H), 7.60–7.64 (m, 3H), 7.75–7.84 (m, 4H), 10.54 (s, 1H), 12.69 (s, 1H); ^13^C NMR (100 MHz, DMSO-*d*_*6*_): *δ* ppm 108.17, 118.82, 121.76, 124.42, 127.06, 127.89, 129.09, 130.07, 134.54, 136.46, 141.07, 142.44, 163.97, 168.76; HRMS (EI) *m/z*:calcd [M]^+^, 385.0555 (C_18_H_15_N_3_O_3_S_2_^+^), found [M]^+^, 385.0559 (C_18_H_15_N_3_O_3_S_2_^+^).

#### *N*-(3-Chloro-4-fluorophenyl)cinnamamide (Dk)

The pure compound was obtained as white powder (yield 90%). Mp: 138–139°C (EtOH); ^1^H NMR (400 MHz, DMSO-*d*_*6*_): *δ* ppm 6.77 (d, *J* = 15.6 Hz, 1H), 7.36–7.46 (m, 4H), 7.52–7.56 (m, 1H), 7.59–7.64 (m, 3H), 8.04 (dd, *J* = 7, 2.4 Hz, 1H), 10.42 (s, 1H); ^13^C NMR (100 MHz, DMSO-*d*_*6*_): *δ* ppm 116.96, 117.17, 119.12, 119.30, 119.47, 119.54, 120.57, 121.63, 127.85, 129.07, 130.03, 134.52, 136.51, 136.54, 140.85, 151.95, 154.36, 163.74; HRMS (EI) *m/z*:calcd [M]^+^, 275.0513 (C_15_H_11_ClFNO^+^), found [M]^+^, 275.0514 (C_15_H_11_ClFNO^+^).

#### (E)-*N*-(4-Chloro-2-fluorophenyl)-3-(2-chlorophenyl)acrylamide (Ec)

The pure compound was obtained as white powder (yield 36%). Mp: 164–165°C (EtOH); ^1^H NMR (400 MHz, DMSO-*d*_*6*_): *δ* ppm 6.59 (d, *J* = 16 Hz, 1H), 7.12 (d, *J* = 16 Hz, 1H), 7.43–7.45 (m, 2H), 7.50–7.56 (m, 1H), 7.74–7.77 (m, 1H), 7.85–7.90 (m, 2H), 8.16 (t, *J* = 8.6 Hz, 1H), 10.15 (s, 1H); ^13^C NMR (100 MHz, DMSO-*d*_*6*_): *δ* ppm 116.05, 116.28, 122.32, 124.41, 124.64, 125.45, 125.57, 127.74, 127.79, 127.92, 128.26, 129.97, 130.13, 131.43, 131.72, 131.85, 132.39, 133.58, 136.11, 138.71, 163.53, 167.21; HRMS (ESI) *m/z*:calcd [M]^+^, 309.0123 (C_15_H_10_Cl_2_FNO^+^), found [M-H]^+^, 308.0055 (C_15_H_9_Cl_2_FNO^+^).

#### (E)-*N*-(3-Chloro-4-fluorophenyl)-3-(2-chlorophenyl)acrylamide (Ek)

The pure compound was obtained as white powder (yield 39%). Mp: 162–163°C (EtOH); ^1^H NMR (400 MHz, DMSO-*d*_*6*_): *δ* ppm 6.59 (d, *J* = 16 Hz, 1H), 6.84 (d, *J* = 15.6 Hz, 1H), 7.40–7.46 (m, 2H), 7.52–7.57 (m, 1H), 7.76–7.78 (m, 1H), 7.85–7.93 (m, 2H), 8.04–8.06 (m, 1H),10.56 (s, 1H); ^13^C NMR (100 MHz, DMSO-*d*_*6*_): *δ* ppm 117.02, 117.23, 119.60, 120.71, 122.32, 124.77, 127.80, 127.99, 128.26, 129.98, 130.14, 131.44, 131.73, 131.85, 132.32, 133.55, 136.33, 138.71, 163.23, 167.20; HRMS (ESI) *m/z*:calcd [M]^+^, 309.0123 (C_15_H_10_Cl_2_FNO^+^), found [M-H]^+^, 308.0051 (C_15_H_9_Cl_2_FNO^+^).

#### *N*-(4-Chloro-2-fluorophenyl)-3-phenylpropanamide (Fc)

The pure compound was obtained as white powder (yield 79%). Mp: 110–111°C (EtOH); ^1^H NMR (400 MHz, DMSO-*d*_*6*_): *δ* ppm 2.69 (t, *J* = 8 Hz, 2H), 2.89 (t, *J* = 8 Hz, 2H), 7.15–7.27 (m, 6H), 7.45 (dd, *J* = 10.8, 2.4 Hz, 1H), 7.91 (t, *J* = 8.8 Hz, 1H), 9.80 (s, 1H); ^13^C NMR (100 MHz, DMSO-*d*_*6*_): *δ* ppm 115.99, 116.22, 124.47, 124.50, 124.99, 125.39, 125.51, 125.99, 127.90, 127.99, 128.29, 128.33, 141.04, 152.07, 154.54, 171.01; HRMS (EI) *m/z*:calcd [M]^+^, 277.0670 (C_15_H_13_ClFNO^+^), found [M]^+^, 277.0665 (C_15_H_13_ClFNO^+^).

#### 3-Phenyl-*N*-(3-(Trifluoromethyl)phenyl)propanamide (Ff)

The pure compound was obtained as orange powder (yield 83%). Mp: 141–142°C (EtOH); ^1^H NMR (400 MHz, DMSO-*d*_*6*_): *δ* ppm 2.65 (t, *J* = 8 Hz, 2H), 2.91 (t, *J* = 8 Hz, 2H), 7.15–7.19 (m, 1H), 7.23–7.28 (m, 4H), 7.36 (d, *J* = 8 Hz, 1H), 7.52 (t, *J* = 8 Hz, 1H), 7.74 (d, *J* = 8.4 Hz, 1H), 8.08 (s, 1H), 10.25 (s, 1H); ^13^C NMR (100 MHz, DMSO-*d*_*6*_): *δ* ppm 114.99, 115.03, 119.37, 119.41, 122.52, 125.49, 126.02, 128.26, 128.37, 129.27, 129.58, 129.99, 139.95, 141.03, 171.03; HRMS (EI) *m/z*:calcd [M]^+^, 293.1027 (C_16_H_14_F_3_NO^+^), found [M]^+^, 293.1034 (C_16_H_14_F_3_NO^+^).

#### 3-Phenyl-*N*-(4-(*N*-(thiazol-2-yl)sulfamoyl)phenyl)propanamide (Fj)

The pure compound was obtained as white powder (yield 88%). Mp: 228–229°C (EtOH); ^1^H NMR (400 MHz, DMSO-*d*_*6*_): *δ* ppm 2.64 (t, *J* = 7.6 Hz, 2H), 2.91 (t, *J* = 8 Hz, 2H), 6.81 (d, *J* = 4.8 Hz, 1H), 7.14–7.28 (m, 6H), 7.68–7.73 (m, 4H), 10.24 (s, 1H), 12.66 (s, 1H); ^13^C NMR (100 MHz, DMSO-*d*_*6*_): *δ* ppm 108.11, 118.53, 124.39, 126.02, 126.98, 128.26, 128.37, 136.15, 141.03, 142.37, 168.72, 171.01; HRMS (EI) *m/z*:calcd [M]^+^, 387.0711 (C_18_H_17_N_3_O_3_S_2_^+^), found [M]^+^, 387.0713 (C_18_H_17_N_3_O_3_S_2_^+^).

#### *N*-(3,4-Difluorophenyl)-2-phenoxyacetamide (Gb)

The pure compound was obtained as white powder (yield 68%). Mp: 90–91°C (EtOH); ^1^H NMR (400 MHz, DMSO-*d*_*6*_): *δ* ppm 4.69 (s, 2H), 6.95–7.02 (m, 2H), 7.37–7.41 (m, 2H), 7.78–7.83 (m, 1H), 10.30 (s, 1H); ^13^C NMR (100 MHz, DMSO-*d*_*6*_): *δ* ppm 108.68, 108.90, 114.72, 116.07 116.10, 116.13, 116.16, 117.40, 117.58, 121.33, 129.58, 125.36, 135.39, 135.48, 144.27, 144.40, 146.68, 146.81, 147.62, 147.75, 150.04, 150.17, 157.72, 167.01; HRMS (EI) *m/z*:calcd [M]^+^, 263.0758 (C_14_H_11_F_2_NO_2_^+^), found [M]^+^, 263.0755 (C_14_H_11_F_2_NO_2_^+^).

#### *N*-(4-Chloro-2-fluorophenyl)-2-phenoxyacetamide (Gc)

The pure compound was obtained as white crystal (yield 84%). Mp: 110–111°C (EtOH); ^1^H NMR (400 MHz, DMSO-*d*_*6*_): *δ* ppm 4.76 (s, 2H), 6.95–6.99 (m, 3H), 7.26–7.33 (m, 2H), 7.51 (dd, *J* = 10.4, 2.4 Hz, 1H), 7.84 (t, *J* = 8.8 Hz, 1H), 9.97 (s, 1H); ^13^C NMR (100 MHz, DMSO-*d*_*6*_): *δ* ppm 115.06, 116.61, 116.84, 121.68, 124.99, 125.04, 125.07, 125.11, 126.16, 129.34, 129.44, 129.98, 153.16, 155.65, 158.13, 167.15; HRMS (EI) *m/z*:calcd [M]^+^, 279.0462 (C_14_H_11_ClFNO_2_^+^), found [M]^+^, 279.0467 (C_14_H_11_ClFNO_2_^+^).

#### 2-Phenoxy-*N*-(4-sulfamoylphenyl)acetamide (Gd)

The pure compound was obtained as white powder (yield 77%). Mp: 206–207°C (EtOH); ^1^H NMR (400 MHz, DMSO-*d*_*6*_): *δ* ppm 3.37 (s, 2H), 4.74 (s, 2H), 6.95–7.01 (m, 3H), 7.27–7.33 (m, 2H), 7.77–7.83 (m, 4H), 10.42 (s, 1H); ^13^C NMR (100 MHz, DMSO-*d*_*6*_): *δ* ppm 67.07, 114.70, 119.31, 121.31, 126.73, 129.59, 138.84, 141.35, 157.77, 167.27; HRMS (EI) *m/z*:calcd [M]^+^, 306.0674 (C_14_H_14_N_2_O_4_S^+^), found [M]^+^, 306.0680 (C_14_H_14_N_2_O_4_S^+^).

#### 2-Phenoxy-*N*-(3-(trifluoromethyl)phenyl)acetamide (Gf)

The pure compound was obtained as orange powder (yield 87%). Mp: 93–94°C (EtOH); ^1^H NMR (400 MHz, DMSO-*d*_*6*_): *δ* ppm 4.72 (s, 2H), 6.95–7.01 (m, 3H), 7.29–7.33 (d, *J* = 9.2 Hz, 1H), 7.43 (d, *J* = 8 Hz, 1H), 7.56 (t, *J* = 8 Hz, 1H), 7.88 (d, *J* = 8 Hz, 1H), 8.12 (s, 1H), 10.41 (s, 1H); ^13^C NMR (100 MHz, DMSO-*d*_*6*_): *δ* ppm 114.69, 115.81, 120.05, 120.09, 121.29, 123.28, 125.44, 129.28, 129.56, 130.04, 139.17, 157.72, 167.31; HRMS (EI) *m/z*:calcd [M]^+^, 295.0820 (C_15_H_12_F_3_NO_2_^+^), found [M]^+^, 295.0822 (C_15_H_12_F_3_NO_2_^+^).

#### *N*-(4-(*N*-(4-Methylpyrimidin-2-yl)sulfamoyl)phenyl)-2-phenoxyacetamide (Gg)

The pure compound was obtained as orange powder (yield 72%). Mp: 249–250°C (EtOH); ^1^H NMR (400 MHz, DMSO-*d*_*6*_): *δ* ppm 3.35 (s, 3H), 4.72 (s, 2H), 6.88 (d, *J* = 5.2 Hz, 1H), 6.93–6.98 (m, 3H), 7.27–7.31 (m, 2H), 7.80 (d, *J* = 8.8 Hz, 2H), 7.94 (d, *J* = 8.8 Hz, 2H), 8.30 (d, *J* = 5.2 Hz, 1H), 10.47 (s, 1H), 11.67 (s, 1H); ^13^C NMR (100 MHz, DMSO-*d*_*6*_): *δ* ppm 23.27, 66.99, 114.63, 118.84, 121.26, 129.78, 129.56, 134.75, 142.22, 156.55, 157.74, 167.34; HRMS (EI) *m/z*:calcd [M]^+^, 398.1049 (C_19_H_18_N_4_O_4_S^+^), found [M]^+^, 398.1049 (C_19_H_18_N_4_O_4_S^+^).

#### *N*-(3-Chloro-4-fluorophenyl)-2-phenoxyacetamide (Gk)

The pure compound was obtained as white powder (yield 69%). Mp: 102–103°C (EtOH); ^1^H NMR (400 MHz, DMSO-*d*_*6*_): *δ* ppm 4.69 (s, 2H), 6.95–7.01 (m, 3H), 7.29–7.33 (m, 2H), 7.38 (t, *J* = 9.2 Hz, 1H), 7.55–7.59 (m, 1H), 7.95 (dd, *J* = 7, 2.8 Hz, 1H), 10.28 (s, 1H); ^13^C NMR (100 MHz, DMSO-*d*_*6*_): *δ* ppm 114.71, 116.85, 117.07, 119.03, 119.21, 120.09, 120.16, 121.22, 121.31, 129.56, 131.60, 135.63, 152.17, 154.59, 157.69, 167.01; HRMS (EI) *m/z*:calcd [M]^+^, 279.0462 (C_14_H_11_ClFNO_2_^+^), found [M]^+^, 279.0439 (C_14_H_11_ClFNO_2_^+^).

### X-ray Crystallography

A single crystal of suitable size for X-ray diffractometry was selected under a microscope and mounted on the tip of a glass fibre, which was positioned on a copper pin. Crystallographic assay was performed as described in the reported protocol [26, 27]. The X-ray data for HS-Dc and HS-Gc were collected with a BRUKER Kappa CCD diffractometer, employing graphite-monochromated Mo-*K*α radiation at 200 K and the *θ*–2*θ* scan mode. The space group for HS-Dc and HS-Gc were determined on the basis of systematic absences and intensity statistics, and the structure of HS-Dc and HS-Gc were solved by direct methods using SIR92 or SIR97 and refined with SHELXL-97.

### Ethics Statement

Human cartilage from OA patients who received total knee joint replacement were obtained aseptically with prior approval of the Institutional Review Board (IRB), Tri-Service General Hospital (Permit Number 1-102-05-091). This study was conducted under the guidelines of the Helsinki Declaration and approved by the Human Subjects Protection Offices (IRB) at the Tri-Service General Hospital. Because all participants provided their written consent to participate in this study, informed consent was obtained by the approving IRB (TSGHIRB No. 1-102-05-091). In this study, we chose the Taiwan Black Pig (approximate age 8–10 months) as breeding pigs from Taoyuan farm in Taiwan. All pigs were sacrificed for routine farm purposes. These breeding pigs were euthanized by electrocution. The isolation and culture of sampling chondrocytes were obtained from the hind leg joints of pigs (n = 16) for all experiments. The care of the pigs, and all procedures were performed according to institutional guidelines, and were approved by the Ethics Committee of Council of Agriculture, Executive Yuan, R.O.C. (Taiwan).

### Isolation and Culture of Chondrocytes

The porcine cartilages were obtained from the hind leg joints of pigs. The preparation of chondrocytes from cartilage was performed according to our previous report [25]. Briefly, the extracted cartilages were firstly minced into small pieces and chondrocytes were isolated by enzymatic digestion of articular cartilage with 2 mg/mL protease in serum-free Dulbecco’s modified Eagle’s medium (DMEM)/antibiotics, the specimens were then digested overnight with 2 mg/mL collagenase I and 0.9 mg/mL hyaluronidase in DMEM containing 10% fetal bovine serum (FBS). The cells were collected, passed through a cell strainer (Becton Dickinson, Mountain View, CA, USA), and cultured in DMEM containing 10% FBS and antibiotics for 3–4 days before use.

Human cartilage from OA patients who received total knee joint replacement were obtained aseptically with prior approval of the Institutional Review Board, Tri-Service General Hospital (TSGHIRB 1-102-05-091). The preparation of first passage chondrocytes from cartilage was performed according to the previous studies [28, 29]. In brief, the full thickness articular cartilage was removed from the underlying bone and cut into pieces of around 0.5 cm^2^. After enzymatic digestion with 2 mg/mL Pronase (Calbiochem, La Jolla, CA) in serum-free DMEM/antibiotics (Gibco-BRL, Gaithersberg, MD) for 1 h at 37°C in 5% CO_2_ atmosphere, the specimens were then digested with collagenase I at 0.25 mg/mL in DMEM medium containing 5% FBS for overnight. Finally, the cells in monolayer culture were suspended and cultured in DMEM medium containing 10% fetal bovine serum and antibiotics for 5–7 days before use.

### Cell Viability Assay

The cytotoxic effects of synthesized compounds were evaluated by using 3-[4,-dimethylthiazol-2-y]-2,5-diphenyl-tetrazolium bromide (MTT) assay [30]. In brief, chondrocytes were seeded into 24-well culture plate at a density of 5×10^4^ per well and then were incubated in the presence or absence of tested compounds for 24 h. Then, 100 μL of MTT (5 mg/mL in H_2_O) was added, and cells were incubated at 37°C for 6 h followed by the addition of 100 μL of DMSO. After incubation at 37°C for another 30 min, the content of dissolved reduced MTT crystals were measured with a plate reader (TECAN, Grodig, Austria).

### Measurement of NO concentrations

The measurement of NO release was reflected by determination of its stable end product, nitrite, in supernatants [29, 30]. The Griess reaction was performed with the concentrations of nitrite measured by a spectrophotometer. In brief, an aliquot (100 μL) of culture supernatant was incubated with 50 μL of 0.1% sulfanilamide in 5% phosphoric acid and 50 μL of 0.1% *N*-1-naphthyl-ethylenediamine dihydrochloride. After 10 min of incubation at room temperature, the absorbance was measured at 550 nm wavelength with a plate reader (Tecan, Grodig, Australia).

### Western Blotting Assay

Enhanced chemiluminescence (ECL) Western Blotting (Amersham-Pharmacia, Arlington Heights, IL, USA) was performed as detailed in our previous reports [29, 30]. Briefly, equal amounts of whole cellular extracts were analyzed on 10% sodium dodecyl sulfate-polyacrylamide gel electrophoresis (SDS-PAGE) and transferred to the nitrocellulose filter. For immunoblotting, the nitrocellulose filter was incubated with Tris-buffered saline with 1% Triton X-100 containing 5% nonfat milk for 1 h and then blotted with antibodies against specific proteins for another 2 h at room temperature. After washing with milk buffer, the filter was incubated with rabbit anti-goat IgG or goat anti-rabbit IgG conjugated to horseradish peroxidase at a concentration of 1:5,000 for 30 min. The filter was incubated with the substrate and then exposed to X-ray film (GE Healthcare, Buckinghamshire, UK).

### Nuclear Extract Preparation and Electrophoretic Mobility Shift Assay (EMSA)

Nuclear extract preparation and EMSA analysis were performed as detailed in our previous reports [29, 30]. The oligonucleotides containing NF-κB-binding site (5’-AGT TGA GGG GAC TTT CCC AGG C-3’) and STAT-3-binding site (5’-CAG AAG GAG AAG CCC TTG CC-3’) were purchased from Promega and used as DNA probes. The DNA probes were radiolabeled with [γ-^32^P]ATP using the T4 kinase (Promega). For the binding reaction, the radiolabeled probe was incubated with 4 μg of nuclear extracts. The binding buffer contained 10 mM Tris–HCl (pH = 7.5), 50 mM NaCl, 0.5 mM ethylenediaminetetraacetic acid (EDTA), 1 mM dithiothreitol, 1 mM MgCl_2_, 4% glycerol, and 2 μg poly(dI-dC). The reaction mixture was left at room temperature to proceed with binding reaction for 20 min. The final reaction mixture was analyzed in a 6% non-denaturing polyacrylamide gel with 0.5×Tris/ borate/EDTA as an electrophoresis buffer.

## Results and Discussion

### Chemical Synthesis

**Fig 3:**
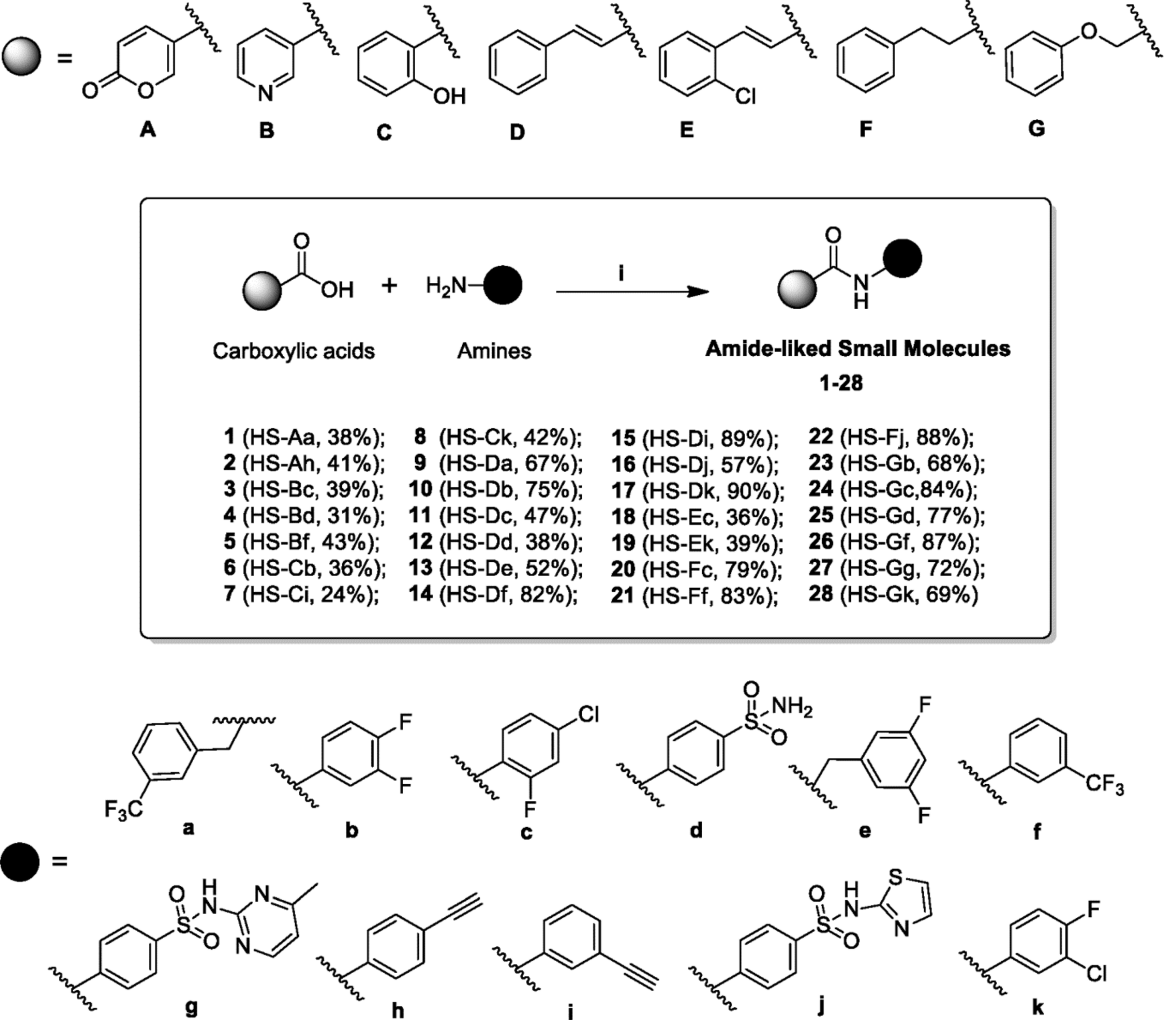
The application of parallel synthesis strategy for the preparation of amide-linked small molecules. Reagents and conditions: (i) HOBt, EDC, DCM, rt.

**Fig 4:**
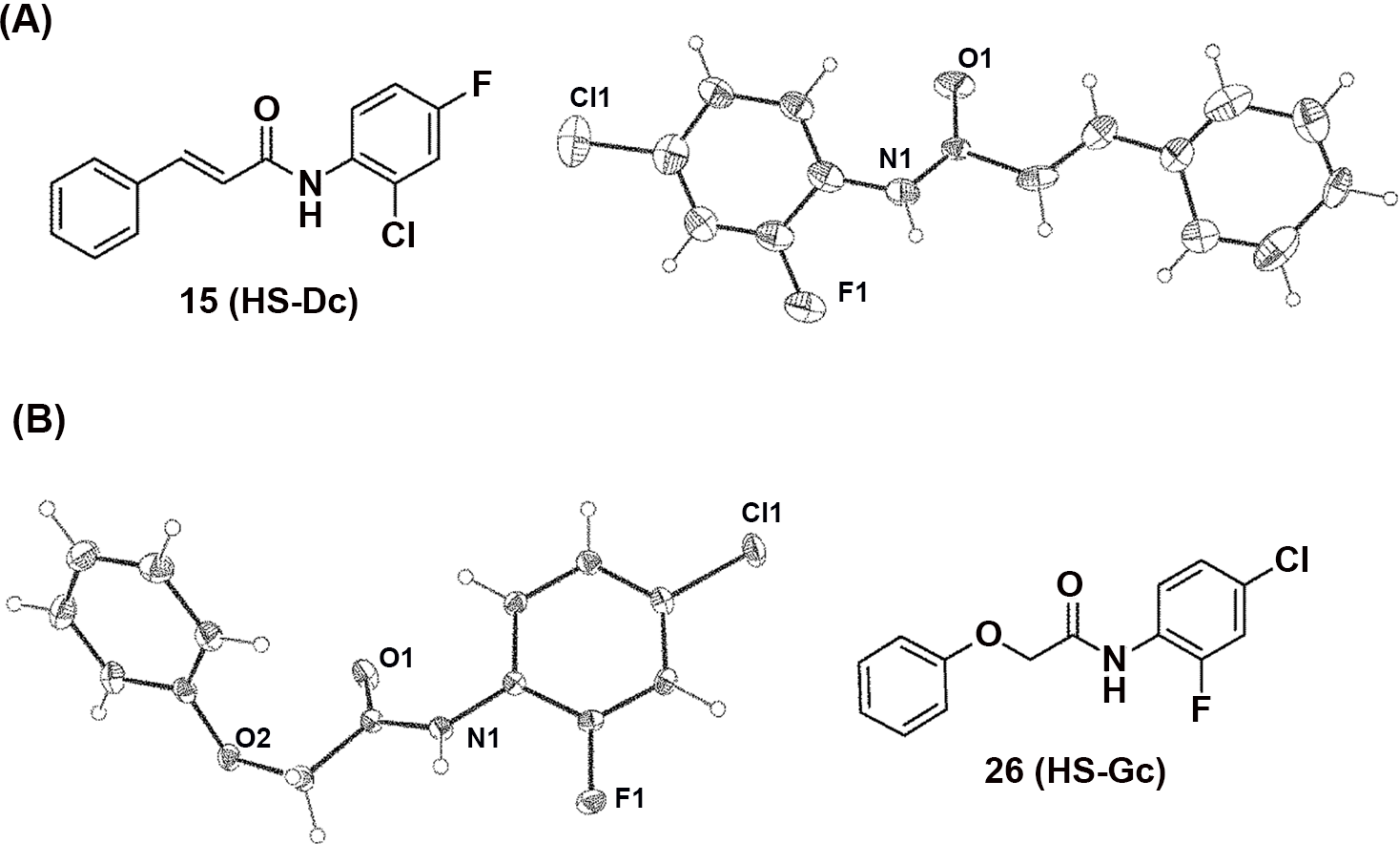
X-ray structures of the synthesized compounds (HS-Dc and HS-Gc).

In this study, the parallel synthesis was successful used to synthesize a diversity of amide-linked small molecules. In the present work, several carboxylic acids, including coumalic acid [31], salicylic acid [32], nicotinic acid [33], cinnamic acid [34], 2-chlorocinnamic acid [35], hydrocinamic acid [36], and 2-phenoxyacetic acid [37], have been found to have wide variety of biological activities. Therefore, these carboxylic acids were selected as our core structures for the preparation of targeted compounds. The synthetic procedures were carried out by coupling the carboxylic acids with the appropriate amines in the presence of 1-hydroxybenzotriazole (HOBt), *N*-ethyl-*N*’-(3-dimethylaminopropyl)carbodiimide (EDC), and dichloromethane (DCM) at room temperature for 3 days (Fig 3). The byproducts were further separated and purified by employing their different physicochemical properties using recrystallization and chromatography procedures. The structures of all synthesized compounds are elucidated along with their spectroscopic characterizations in the experimental section. Furthermore, two synthesized structures (HS-Dc and HS-Gc) were confirmed by using the single-crystal X-ray crystallography (Fig 4).

### Effects of all Synthesized Compounds on TNF-α-induced NO Production in Chondrocytes

**Fig 5:**
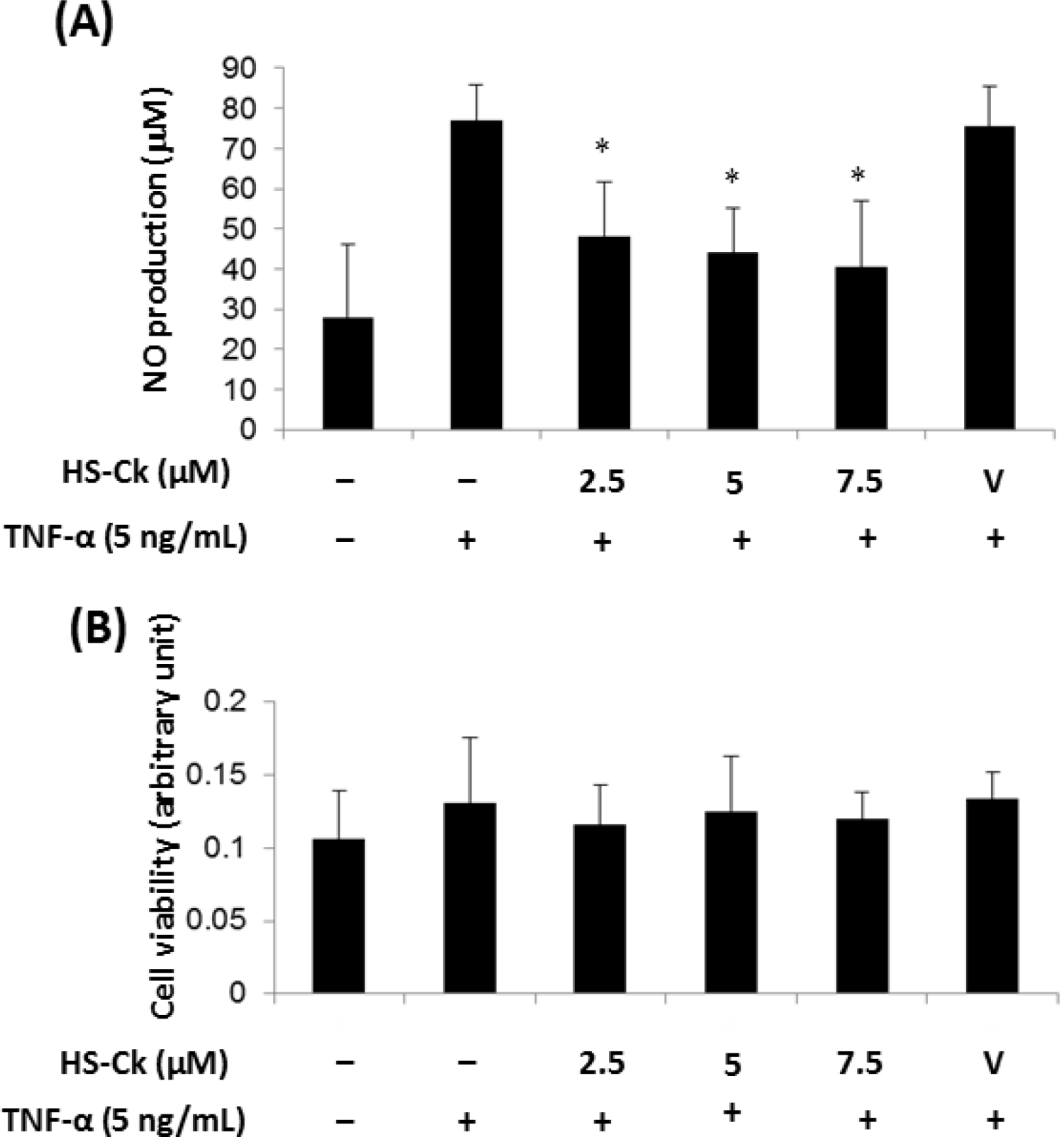
Effects of HS-Ck on TNF-α-induced NO production and cell viability in porcine chondrocytes. (A) Porcine chondrocytes were pretreated with various doses of HS-Ck or the DMSO solvent as vehicle (V) control for 2 h and then stimulated with TNF-α for 24 h. The production of NO was determined by using the Griess reagent. (B) To determine potential cytotoxic effects of HS-Ck, porcine chondrocytes were treated with various concentrations of HS-Ck for 24 h. The cells and culture supernatants were collected and determined by using MTT assay. The representative data out of at least three independent experiments are shown. *P < 0.05 compared to the TNF-α-stimulated in the absence of HS-Ck treatment.

In order to evaluate the effects of all synthesized compounds on anti-inflammatory activities, TNF-α-induced NO production in porcine chondrocytes are used as a screening model in this study. As shown in Table 1, the highest percent inhibition values of NO production were for three compounds **8** (HS-Ck), **11** (HS-Dc), and **6** (HS-Cb) at 10 μM (78.66 ± 0.21%, 50.24 ± 0.50%, and 43.16 ± 0.57%, respectively). Further, the most potent compound HS-Ck was found to dose-dependently suppress TNF-α-induced NO production in porcine chondrocytes with IC_50_ value of 7.15 ± 2.25 μM. The inhibitory effects of HS-Ck on TNF-α-induced NO production in porcine chondrocytes were significantly suppressed at concentrations of 2.5, 5, and 7.5 μM (Fig 5A). To investigate whether HS-Ck did not affect the viability of porcine chondrocytes, MTT assay was used for identifying the cell survival rate. The observation of HS-Ck proves that its inhibitory activity was not due to its cytotoxic effect in porcine chondrocytes (Fig 5B).

**Table 1:** Effects of all synthesized compounds on TNF-α-induced NO production in porcine chondrocytes.

Compound	Name	Inhibition of NO production (%) ± SD^a^
**1**	HS-Aa	0 ± 1.02
**2**	HS-Ah	0 ± 1.14
**3**	HS-Bc	4.06 ± 0.96
**4**	HS-Bd	10.24 ± 0.90
**5**	HS-Bf	3.48 ± 0.97
**6**	HS-Cb	43.16 ± 0.57
**7**	HS-Ci	38.28 ± 0.61
**8**	HS-Ck	78.66 ± 0.21
**9**	HS-Da	35.94 ± 0.64
**10**	HS-Db	11.79 ± 0.88
**11**	HS-Dc	50.24 ± 0.50
**12**	HS-Dd	9.08 ± 0.91
**13**	HS-De	10.82 ± 0.89
**14**	HS-Df	2.51 ± 0.97
**15**	HS-Di	13.53 ± 0.86
**16**	HS-Dj	5.80 ± 0.94
**17**	HS-Dk	15.46 ± 0.85
**18**	HS-Ec	23.19 ± 0.77
**19**	HS-Ek	10.68 ± 0.89
**20**	HS-Fc	4.79 ± 0.95
**21**	HS-Ff	0.87 ± 0.99
**22**	HS-Fj	0 ± 1.05
**23**	HS-Gb	0 ± 1.12
**24**	HS-Gc	0 ± 1.16
**25**	HS-Gd	5.88 ± 0.82
**26**	HS-Gf	1.09 ± 0.99
**27**	HS-Gg	9.59 ± 0.91
**28**	HS-Gk	1.96 ± 0.98

^*a*^ Porcine chondrocytes were pretreated with all synthesized compounds at 10 μM for 2 h, and then stimulated with TNF-α (5 ng/mL) for 24 h. The amount of nitrite with TNF-α-treated only group was set as 100.0%. Inhibition (%) = 100%—NO production (%) of compounds. NO concentration in medium was determined using the Griess reagent. All representative data were performed the means ± SD at least three times independently (*n* > 3).

**Fig 6:**
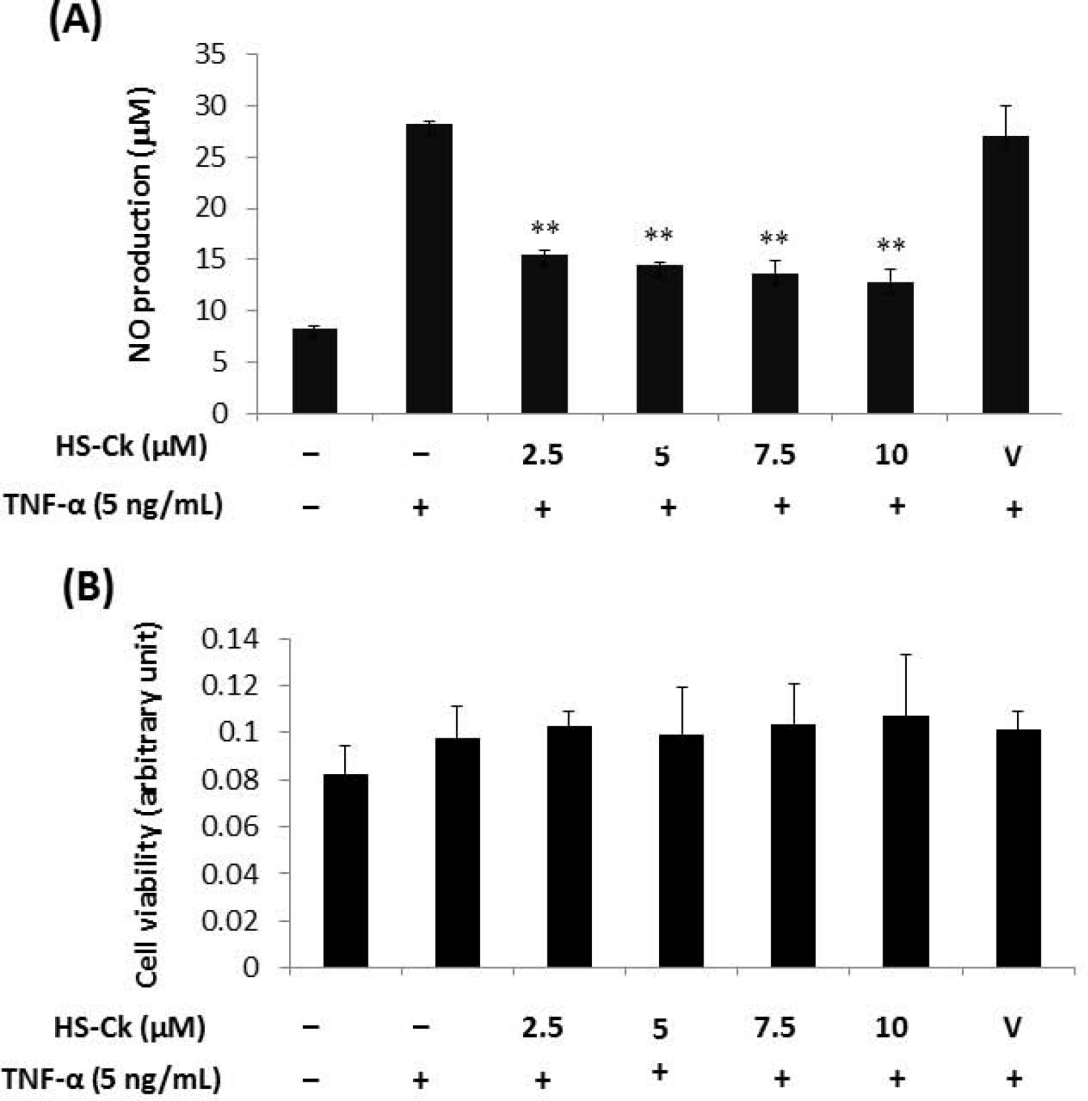
Effects of HS-Ck on TNF-α-induced NO production and cell viability in human chondrocytes. (A) Human chondrocytes were pretreated with various doses of HS-Ck or the DMSO solvent as vehicle (V) control for 2 h and then stimulated with TNF-α for 24 h. The production of NO was determined by using the Griess reagent. (B) To determine potential cytotoxic effects of HS-Ck, human chondrocytes were treated with various concentrations of HS-Ck for 24 h. The cells and culture supernatants were collected and determined by using MTT assay. The representative data out of at least three independent experiments are shown. **P < 0.01 compared to the TNF-α-stimulated in the absence of HS-Ck treatment.

In addition, the inhibitory effects of HS-Ck on TNF-α-induced NO production in human chondrocytes were significantly suppressed at concentrations of 2.5, 5, 7.5, and 10 μM (Fig 6A). HS-Ck was also found to dose-dependently decrease TNF-α-induced NO production in human chondrocytes with IC_50_ value of 7.15 ± 0.25 μM. Interestingly, the observation of HS-Ck proves that its inhibitory activity was also not due to its cytotoxic effect in human chondrocytes (Fig 6B).

Based on these primary results of TNF-α-induced NO releases, the SARs of all synthesized compounds are briefly summarized as follows. Amongst compounds bearing the carboxylic acid moieties, we found that the salicylic acid skeleton showed relatively higher anti-inflammatory activities than the others. Amongst compounds bearing the di-substituted groups at the aniline moieties, we observed that these compounds showed relatively higher activities than the mono-substituted groups. On the basis of TNF-α-induced NO production results, we conclude that *N*-(3-chloro-4-fluorophenyl)-2-hydroxybenzamide (HS-Ck) is the most potent anti-inflammatory and/or immunomodulatory agent for further study. Thus, we chose HS-Ck that was the most potent among the synthesized series for the mechanistic investigation in TNF-α-induced inflammatory signaling pathway.

### Effects of HS-Ck on TNF-α-induced Expression Levels of iNOS and MMP-13 in Porcine Chondrocytes

**Fig 7:**
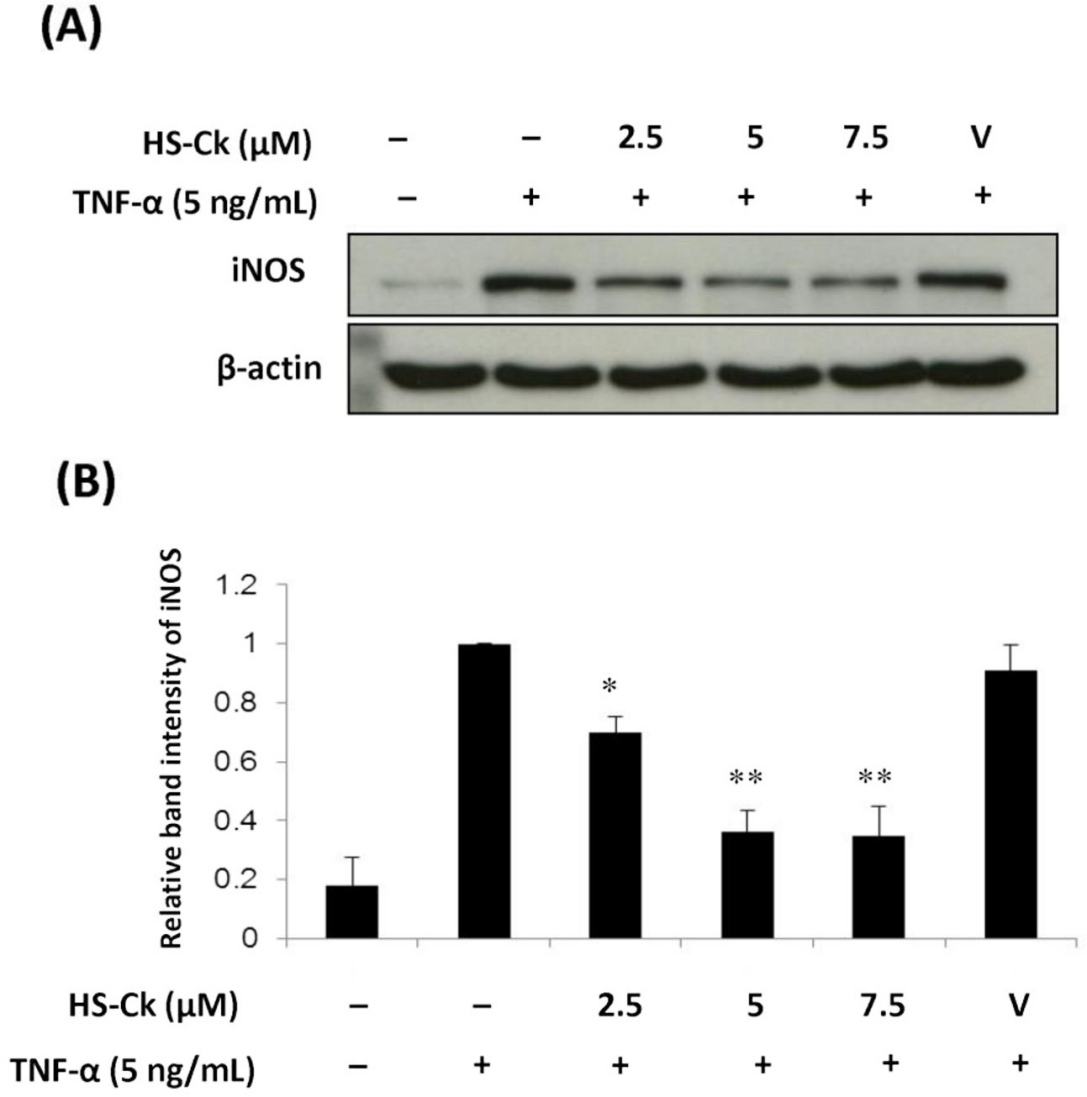
Effects of HS-Ck on the expression levels of iNOS in TNF-α-induced porcine chondrocytes. (A) Porcine chondrocytes were pretreated with various doses of HS-Ck or the DMSO solvent as vehicle (V) control for 2 h and then stimulated with TNF-α for 24 h. The expression levels of iNOS and β-actin were determined by using Western Blotting assay. (B) The relative band intensity of iNOS was normalized as the corresponding the band of β-actin. The representative data out of at least three independent experiments are shown. *P < 0.05 and **P < 0.01 compared to the TNF-α-stimulated in the absence of HS-Ck treatment.

The accumulating evidences showed that the inflammatory cytokine TNF-α contribute to an increased NO production and the activation of inducible nitric oxide synthase (iNOS) pathway [38, 39]. Moreover, the iNOS-NO system has been demonstrated to be the essential factor in OA pathogenesis [40–42]. Therefore, we further determine whether HS-Ck would suppress the TNF-α-induced expression levels of iNOS in porcine chondrocytes by using Western Blotting assay. As shown in Fig 7, the results showed that the expression levels of iNOS were significantly suppressed after treatment with HS-Ck at concentrations of 2.5, 5, 7.5 μM. Thus, these results revealed that HS-Ck could significantly decrease the TNF-α-induced iNOS expression in porcine chondrocytes.

**Fig 8:**
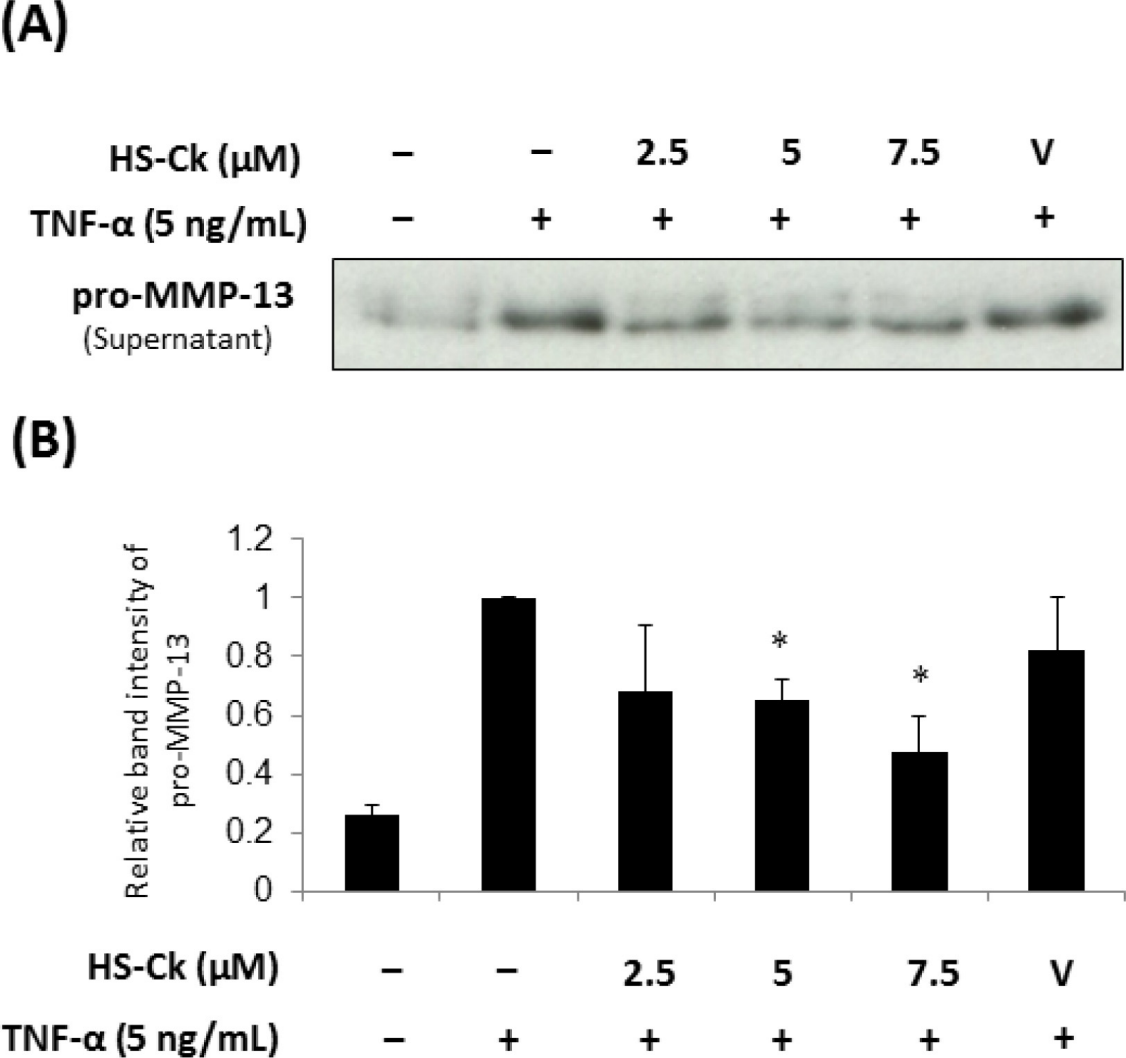
Effects of HS-Ck on the expression levels of MMP-13 in TNF-α-induced porcine chondrocytes. (A) Porcine chondrocytes were pretreated with various doses of HS-Ck or the DMSO solvent as vehicle (V) control for 2 h and then stimulated with TNF-α (5 ng/mL) for 24 h. The activities of pro-MMP-13 released into the culture supernatants were determined by using Western Blotting assay. (B) The expression levels of pro-MMP-13 activities were expressed as the relative band intensity. The representative data out of at least three independent experiments are shown. *P < 0.05 compared to the TNF-α-stimulated in the absence of HS-Ck treatment.

In addition, the enzymatic cleavage of MMP-13 plays a key role in the pathogenesis of cartilage degradation [17]. Since type II collagen is preferentially cleaved by MMP-13, the accumulating studies showed that MMP-13 can be considered as a principle target for the treatment of OA [43–45]. In order to investigate the chondroprotective effects of HS-Ck, the suppressive effects of HS-Ck on TNF-α-induced pro-MMP-13 expression levels in porcine chondrocytes were further examined by using Western Blotting assay. This assay can analyze the pro-MMP-13 activity through determining the expression levels of TNF-α-induced pro-MMP-13 activity in the culture supernatants. As shown in Fig 8, the results showed that the expression levels of pro-MMP-13 were significantly suppressed after treatment with HS-Ck at concentrations of 5 and 7.5 μM. Altogether, the above results revealed that HS-Ck could significantly suppressive the TNF-α-induced expression levels of iNOS and MMP-13 in porcine chondrocytes.

### Effects of HS-Ck on TNF-α-induced Activation of NF-κB and STAT-3 Transcriptional Factors in Porcine Chondrocytes

Since the activation of NF-κB and STAT-3 transcriptional factors play vital roles in inflammatory responses [10, 11], these transcriptional factors are also important in regulating iNOS and MMP-13 activities [14–16]. In addition, the inflammatory effects of TNF-α can induce different signaling pathway, including NF-κB and STAT-3 activation, which are responsible of increased NO production and MMP-13 activity. To evaluate the chondroprotective effects of HS-Ck on TNF-α-induced activation of NF-κB and STAT-3 transcriptional factors in porcine chondrocytes, we further investigated the suppressive effects of HS-Ck on TNF-α-induced transcriptional activation of NF-κB and STAT-3 by using electrophoretic mobility shift assay (EMSA) for detecting protein–nucleic acid interactions.

**Fig 9:**
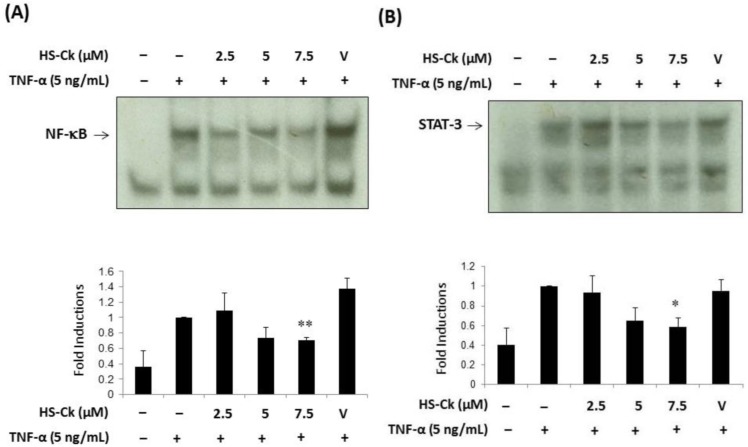
Effects of HS-Ck on TNF-α-stimulated activation of NF-κB and STAT-3 transcriptional factors in porcine chondrocytes. For determining the effects of HS-Ck on TNF-α-induced DNA-binding activity of NF-κB (A) and STAT-3 (B), various doses of HS-Ck were pretreated with nuclear extracts for 30 min before the addition of radiolabeled oligonucleotides. The effects of HS-Ck on TNF-α-induced DNA-binding activity of NF-κB and STAT-3 were determined by using EMSA. The expression levels of NF-κB (A) and STAT-3 (B) were expressed as the relative band intensity. DMSO solvent was showed as vehicle (V) control. The representative data out of at least three independent experiments are shown. **P < 0.01 compared to the TNF-α-stimulated in the absence of HS-Ck treatment.

As shown in Fig 9, the expression levels of NF-κB and STAT-3 were increased by TNF-α treatment. However, such elevated levels were reduced by treatment with HS-Ck at 7.5 μM. Our results showed that the expression levels of NF-κB and STAT-3 were significantly suppressed after treatment with HS-Ck at 7.5 μM. Thus, the mechanism of action of HS-Ck might reduce TNF-α-induced inflammatory responses through suppressing the activation of NF-κB and STAT-3 transcriptional factors in this study.

## Conclusion

**Fig 10:**
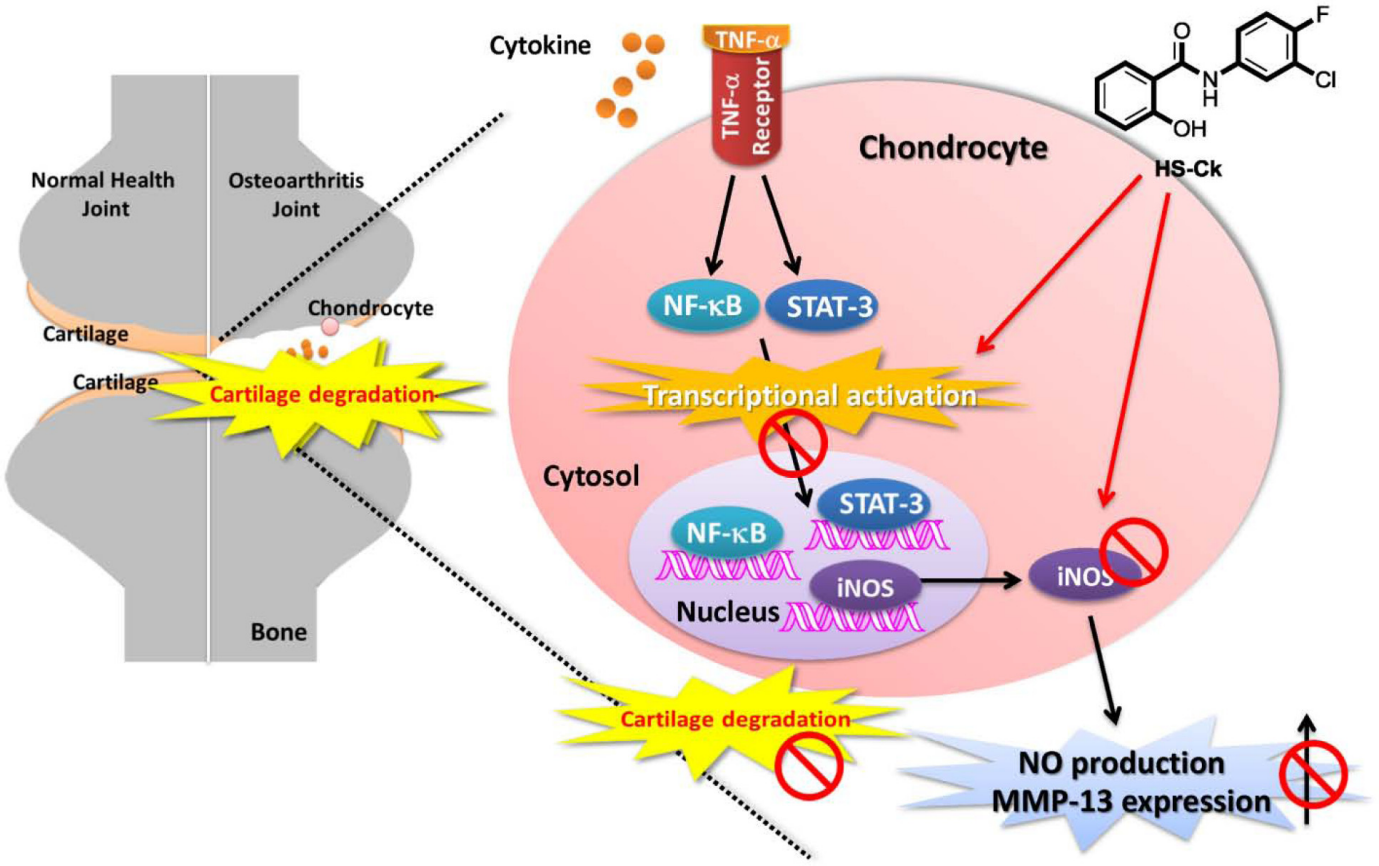
Proposed mechanism of action for the chondroprotective effects of HS-Ck on TNF-α-induced cartilage degradation in porcine chondrocytes includes the suppressive effects on activation of NF-κB and STAT-3 transcriptional factors, iNOS expression, NO production, and MMP-13 activities.

In a continuous interest in the development of anti-inflammatory agents, the parallel synthesis strategy was used for the development of a new class of chondroprotective agents in osteoarthritis therapeutics. In the present study, bioassay screening of amide-linked small molecules revealed that HS-Ck was the most potent inhibitor of NO production and iNOS expression in TNF-α-induced chondrocytes. In addition, our biological results indicated that HS-Ck a potent chondroprotective agent toward TNF-α-induced cartilage degradation. The proposed mechanism of this study is showed in Fig 10. The chondroprotective effects of HS-Ck might decrease iNOS-NO production and MMP-13 activity through suppressing the activation of NF-κB and STAT-3 transcriptional factors. Thus, HS-Ck could be considered as a new potent chondroprotective agents in OA therapeutics.

## Supporting Information

S1 Fig Spectroscopic characterizations (^1^H NMR, ^13^C NMR, and HRMS spectra) of HS-Ck. (DOCX)

## References

[1] Felson DT. Clinical practice. Osteoarthritis of the knee. N. Engl. J. Med. 2006; 354:841–8. 10.1056/NEJMcp051726 . 16495396

[2] Lane NE. Clinical practice. Osteoarthritis of the hip. N. Engl. J. Med. 2007; 357:1413–21. 10.1056/NEJMcp071112 . 17914042

[3] Yoshihara Y, Nakamura H, Obata K, Yamada H, Hayakawa T, Fujikawa K, et al Matrix metalloproteinases and tissue inhibitors of metalloproteinases in synovial fluids from patients with rheumatoid arthritis or osteoarthritis. Ann. Rheum. Dis. 2000; 59:455–61. 10834863 10.1136/ard.59.6.455 PMC1753174

[4] Mort JS, Billington CJ. Articular cartilage and changes in arthritis: matrix degradation. Arthritis Res. 2001; 3:337–41. 11714387 10.1186/ar325 PMC128908

[5] Meszaros E, Malemud CJ. Prospects for treating osteoarthritis: enzyme-protein interactions regulating matrix metalloproteinase activity. Ther. Adv. Chronic Dis. 2012; 3:219–29. 10.1177/2040622312454157 23342237 PMC3539270

[6] Goldring MB, Otero M, Plumb DA, Dragomir C, Favero M, El Hachem K, et al Roles of inflammatory and anabolic cytokines in cartilage metabolism: signals and multiple effectors converge upon MMP-13 regulation in osteoarthritis. Eur. Cell Mater. 2011; 21:202–20. 21351054 10.22203/ecm.v021a16 PMC3937960

[7] Magnano MD, Chakravarty EF, Broudy C, Chung L, Kelman A, Hillygus J, et al A pilot study of tumor necrosis factor inhibition in erosive/inflammatory osteoarthritis of the hands. J. Rheumatol. 2007; 34:1323–7. 17516620

[8] Wojdasiewicz P, Poniatowski ŁA, Szukiewicz D. The role of inflammatory and anti-inflammatory cytokines in the pathogenesis of osteoarthritis. Mediat. Inflamm. 2014; 2014:19 10.1155/2014/561459 PMC4021678 24876674

[9] Aktas E, Sener E, Zengin O, Gocun P, Deveci M. Serum TNF-alpha levels: potential use to indicate osteoarthritis progression in a mechanically induced model. Eur. J. Orthop. Surg. Traumatol. 2012; 22:119–22. 10.1007/s00590-011-0803-0

[10] Haseeb A, Haqqi TM. Immunopathogenesis of osteoarthritis. Clin. Immunol. 2013; 146:185–96. 10.1016/j.clim.2012.12.011 23360836 PMC4015466

[11] Marcu KB, Otero M, Olivotto E, Borzi RM, Goldring MB. NF-kappaB signaling: multiple angles to target OA. Curr. Drug Targets. 2010; 11:599–613. 20199390 10.2174/138945010791011938 PMC3076145

[12] Tracey KJ. Physiology and immunology of the cholinergic antiinflammatory pathway. J. Clin. Invest. 2007; 117:289–96. 10.1172/JCI30555 17273548 PMC1783813

[13] Yarilina A, Park-Min K-H, Antoniv T, Hu X, Ivashkiv LB. TNF activates an IRF1-dependent autocrine loop leading to sustained expression of chemokines and STAT1-dependent type I interferon-response genes. Nat. Immunol. 2008; 9:378–87. http://www.nature.com/ni/journal/v9/n4/suppinfo/ni1576_S1.html. 10.1038/ni1576 18345002

[14] Liacini A, Sylvester J, Li WQ, Huang W, Dehnade F, Ahmad M, et al Induction of matrix metalloproteinase-13 gene expression by TNF-alpha is mediated by MAP kinases, AP-1, and NF-kappaB transcription factors in articular chondrocytes. Exp. Cell Res. 2003; 288:208–17. . 12878172 10.1016/s0014-4827(03)00180-0

[15] Goldring M. Osteoarthritis and cartilage: The role of cytokines. Curr. Rheumatol. Rep. 2000; 2:459–65. 10.1007/s11926-000-0021-y 11123098

[16] Tracey D, Klareskog L, Sasso EH, Salfeld JG, Tak PP. Tumor necrosis factor antagonist mechanisms of action: a comprehensive review. Pharmacol. Ther. 2008; 117:244–79. 10.1016/j.pharmthera.2007.10.001 . 18155297

[17] Sabatini M, Lesur C, Thomas M, Chomel A, Anract P, de Nanteuil G, et al Effect of inhibition of matrix metalloproteinases on cartilage loss in vitro and in a guinea pig model of osteoarthritis. Arthritis Rheum. 2005; 52:171–80. 10.1002/art.20900 . 15641085

[18] Gooding OW. Process optimization using combinatorial design principles: parallel synthesis and design of experiment methods. Curr. Opin. Chem. Biol. 2004; 8:297–304. 10.1016/j.cbpa.2004.04.009 . 15183328

[19] Geysen HM, Schoenen F, Wagner D, Wagner R. Combinatorial compound libraries for drug discovery: an ongoing challenge. Nat. Rev. Drug Discov. 2003; 2:222–30. 10.1038/nrd1035 . 12612648

[20] Kolb HC, Sharpless KB. The growing impact of click chemistry on drug discovery. Drug Discov. Today. 2003; 8:1128–37. . 14678739 10.1016/s1359-6446(03)02933-7

[21] Selway CN, Terrett NK. Parallel-compound synthesis: methodology for accelerating drug discovery. Bioorg. Med. Chem. 1996; 4:645–54. . 8804528 10.1016/0968-0896(96)00058-2

[22] Shah N, Gao M, Tsutsui K, Lu A, Davis J, Scheuerman R, et al A novel approach to high-throughput quality control of parallel synthesis libraries. J. Comb. Chem. 2000; 2:453–60. . 11029170 10.1021/cc000011o

[23] Liou JT, Huang HS, Chiang ML, Lin CS, Yang SP, Ho LJ, et al A salicylate-based small molecule HS-Cm exhibits immunomodulatory effects and inhibits dipeptidyl peptidase-IV activity in human T cells. Eur. J. Pharmacol. 2014; 726:124–32. 10.1016/j.ejphar.2014.01.049 . 24491838

[24] Cheng CP, Huang HS, Hsu YC, Sheu MJ, Chang DM. A benzamide-linked small molecule NDMC101 inhibits NFATc1 and NF-kappaB activity: a potential osteoclastogenesis inhibitor for experimental arthritis. J. Clin. Immunol. 2012; 32:762–77. 10.1007/s10875-012-9660-9 . 22396044

[25] Liu FC, Huang HS, Huang CY, Yang R, Chang DM, Lai JH, et al A benzamide-linked small molecule HS-Cf inhibits TNF-alpha-induced interferon regulatory factor-1 in porcine chondrocytes: a potential disease-modifying drug for osteoarthritis therapeutics. J. Clin. Immunol. 2011; 31:1131–42. 10.1007/s10875-011-9576-9 . 21858617

[26] Lee CC, Liu FL, Chen CL, Chen TC, Liu FC, Ahmed Ali AA, et al Novel inhibitors of RANKL-induced osteoclastogenesis: Design, synthesis, and biological evaluation of 6-(2,4-difluorophenyl)-3-phenyl-2H-benzo[e][1,3]oxazine-2,4(3H)-diones. Bioorg. Med. Chem. 2015; 23:4522–32. 10.1016/j.bmc.2015.06.007 . 26081760

[27] Lee CC, Liu FL, Chen CL, Chen TC, Chang DM, Huang HS. Discovery of 5-(2',4'-difluorophenyl)-salicylanilides as new inhibitors of receptor activator of NF-kappaB ligand (RANKL)-induced osteoclastogenesis. Eur. J. Med. Chem. 2015; 98:115–26. 10.1016/j.ejmech.2015.05.015 . 26005025

[28] Forsyth CB, Pulai J, Loeser RF. Fibronectin fragments and blocking antibodies to alpha2beta1 and alpha5beta1 integrins stimulate mitogen-activated protein kinase signaling and increase collagenase 3 (matrix metalloproteinase 13) production by human articular chondrocytes. Arthritis Rheum. 2002; 46:2368–76. 10.1002/art.10502 . 12355484

[29] Ho LJ, Lin LC, Hung LF, Wang SJ, Lee CH, Chang DM, et al Retinoic acid blocks pro-inflammatory cytokine-induced matrix metalloproteinase production by down-regulating JNK-AP-1 signaling in human chondrocytes. Biochem. Pharmacol. 2005; 70:200–8. 10.1016/j.bcp.2005.04.039 . 15946654

[30] Liu FC, Hung LF, Wu WL, Chang DM, Huang CY, Lai JH, et al Chondroprotective effects and mechanisms of resveratrol in advanced glycation end products-stimulated chondrocytes. Arthritis Res. Ther. 2010; 12:R167 10.1186/ar3127 20825639 PMC2990994

[31] Cheng F, Chen W, Huang Y, Zhang C, Shen Y, Hai H, et al Protective effect of areca inflorescence extract on hydrogen peroxide-induced oxidative damage to human serum albumin. Food Rev. Int. 2011; 44:98–102. 10.1016/j.foodres.2010.11.005

[32] Klessig DF, Durner J, Noad R, Navarre DA, Wendehenne D, Kumar D, et al Nitric oxide and salicylic acid signaling in plant defense. Proc. Natl. Acad. Sci. 2000; 97:8849–55. 10.1073/pnas.97.16.8849 10922045 PMC34022

[33] Bogan KL, Brenner C. Nicotinic acid, nicotinamide, and nicotinamide riboside: a molecular evaluation of NAD+ precursor vitamins in human nutrition. annu. Rev. Nutr. 2008; 28:115–30. 10.1146/annurev.nutr.28.061807.155443 . 18429699

[34] Akao Y, Maruyama H, Matsumoto K, Ohguchi K, Nishizawa K, Sakamoto T, et al Cell growth inhibitory effect of cinnamic acid derivatives from propolis on human tumor cell lines. Biol. Pharm. Bull. 2003; 26:1057–9. 10.1248/bpb.26.1057 12843641

[35] Čapková K, Yoneda Y, Dickerson TJ, Janda KD. Synthesis and structure–activity relationships of second-generation hydroxamate botulinum neurotoxin a protease inhibitors. Bioorg. Med. Chem. Lett. 2007; 17:6463–6. 10.1016/j.bmcl.2007.09.103 17951059 PMC2170532

[36] An M, Pratley JE, Haig T. Phytotoxicity of vulpia residues: III. Biological activity of identified allelochemicals from vulpia myuros. J. Chem. Ecol. 2001; 27:383–94. 10.1023/A:1005640708047 14768822

[37] Pan B, Huang R, Zheng L, Chen C, Han S, Qu D, et al Thiazolidione derivatives as novel antibiofilm agents: Design, synthesis, biological evaluation, and structure–activity relationships. Eur. J. Med. Chem. 2011; 46:819–24. 10.1016/j.ejmech.2010.12.014 21255878

[38] Pelletier JP, Martel-Pelletier J. New trends in the treatment of osteoarthritis. Semin. Arthritis Rheum. 2005; 34:13–4. . 16206950 10.1016/j.semarthrit.2004.03.005

[39] Pelletier JP, Martel-Pelletier J, Abramson SB. Osteoarthritis, an inflammatory disease: potential implication for the selection of new therapeutic targets. Arthritis Rheum. 2001; 44:1237–47. 10.1002/1529-0131(200106)44:6<1237::AID-ART214>3.0.CO;2-F . 11407681

[40] Pelletier JP, Jovanovic D, Fernandes JC, Manning P, Connor JR, Currie MG, et al Reduced progression of experimental osteoarthritis in vivo by selective inhibition of inducible nitric oxide synthase. Arthritis Rheum. 1998;41:1275–86. 10.1002/1529-0131(199807)41:7<1275::AID-ART19>3.0.CO;2-T . 9663486

[41] Pelletier JP, Jovanovic DV, Lascau-Coman V, Fernandes JC, Manning PT, Connor JR, et al Selective inhibition of inducible nitric oxide synthase reduces progression of experimental osteoarthritis in vivo: possible link with the reduction in chondrocyte apoptosis and caspase 3 level. Arthritis Rheum. 2000; 43:1290–9. 10.1002/1529-0131(200006)43:6<1290::AID-ANR11>3.0.CO;2-R . 10857787

[42] Tripathi P, Tripathi P, Kashyap L, Singh V. The role of nitric oxide in inflammatory reactions. FEMS Immunol. Med. Microbiol. 2007; 51:443–52. 10.1111/j.1574-695X.2007.00329.x . 17903207

[43] Jungel A, Ospelt C, Lesch M, Thiel M, Sunyer T, Schorr O, et al Effect of the oral application of a highly selective MMP-13 inhibitor in three different animal models of rheumatoid arthritis. Ann. Rheum. Dis. 2010; 69:898–902. 10.1136/ard.2008.106021 19497915 PMC2925150

[44] Li NG, Shi ZH, Tang YP, Wang ZJ, Song SL, Qian LH, et al New hope for the treatment of osteoarthritis through selective inhibition of MMP-13. Curr. Med. Chem. 2011; 18:977–1001. . 21254976 10.2174/092986711794940905

[45] Baragi VM, Becher G, Bendele AM, Biesinger R, Bluhm H, Boer J, et al A new class of potent matrix metalloproteinase 13 inhibitors for potential treatment of osteoarthritis: Evidence of histologic and clinical efficacy without musculoskeletal toxicity in rat models. Arthritis Rheum. 2009; 60:2008–18. 10.1002/art.24629 . 19565489

