# Correction: A New Application of Parallel Synthesis Strategy for Discovery of Amide-Linked Small Molecules as Potent Chondroprotective Agents in TNF-α-Stimulated Chondrocytes

**DOI:** 10.1371/journal.pone.0154067

**Published:** 2016-04-14

**Authors:** The PLOS ONE Staff

The following information is missing from the Funding section: This study was supported by a National Science Council Grant (NSC 102-2314-B-016-049-MY3), the Ministry of Science and Technology (MOST103-2113-M-038-002), Taipei Medical University (TMU102-AE1-B32), and Tri-Service General Hospital (TSGH-C103-070 and TSGH-C104-068). The publisher apologizes for this error.
