# Supplementary material for: A New Application of Parallel Synthesis Strategy for Discovery of Amide-Linked Small Molecules as Potent Chondroprotective Agents in TNF-α-Stimulated Chondrocytes
Source: PLoS One. 2016 Mar 10;11(3):e0149317. doi: 10.1371/journal.pone.0149317 (PMC4786219; doi:10.1371/journal.pone.0149317)
Supplement: S1 Fig — (DOCX) [file pone.0149317.s001.docx]

**< Supporting Information for Plos One** >

**A New Application of Parallel Synthesis Strategy for Discovery of Amide-linked Small Molecules as Potent Chondroprotective Agents in TNF-α-stimulated Chondrocytes**

Chia-Chung Lee^1, 2, 3^, Yang Lo^2^, Ling-Jun Ho^4^, Jenn-Haung Lai^5^, Shiu-Bii Lien^6^, Leou-Chyr Lin^6^, Chun-Liang Chen^1, 2^, Tsung-Chih Chen^1, 2^, Feng-Cheng Liu^3, 7^*, Hsu-Shan Huang^1, 2, 3^*

^1^ Graduate Institute of Cancer Biology and Drug Discovery, College of Medical Science and Technology, Taipei Medical University, Taipei 110, Taiwan

^2^ School of Pharmacy, National Defense Medical Center, Taipei 114, Taiwan

^3^ Graduate Institute of Medical Science, National Defense Medical Center, Taipei 114, Taiwan

^4^ Institute of Cellular and System Medicine, National Health Research Institute, Zhunan 350, Taiwan

^5^ Division of Allergy, Immunology and Rheumatology, Department of Internal Medicine, Chang Gung Memorial Hospital, Chang Gung University, Tao-Yuan 333, Taiwan

^6^ Department of Orthopaedics, Tri-Service General Hospital, National Defense Medical Center, Taipei 114, Taiwan

^7^ Rheumatology/Immunology/Allergy, Tri-Service General Hospital, National Defense Medical Center, Taipei 114, Taiwan

* Corresponding author: Graduate Institute of Cancer Biology and Drug Discovery, College of Medical Science and Technology, Taipei Medical University, Taipei 110, Taiwan; Dr. H.-S. Huang; Tel: +886-2-2736-1661 ext.7525, Fax: [+886-2-6638-7537](tel:%2B886-2-6638-7537), E-mail: [huanghs99@tmu.edu.tw](mailto:huanghs99@tmu.edu.tw);

* Corresponding author: Rheumatology/Immunology/Allergy, Tri-Service General Hospital, National Defense Medical Center, Taipei 114, Taiwan; Dr. F.-C. Liu; Tel: +886-2-8792-3311 ext.12588, Fax: +886-2-8792-7136, E-mail: [lfc10399@yahoo.com.tw](mailto:lfc10399@yahoo.com.tw)

**Contents of Supporting Information**

**Page**

1. Spectral data of HS-Ck…….….............................................................................................**S2-S4**

**S1 Fig.** ^1^H NMR (A) ^13^C NMR (B) and HRMS (C) spectra of HS-Ck

**
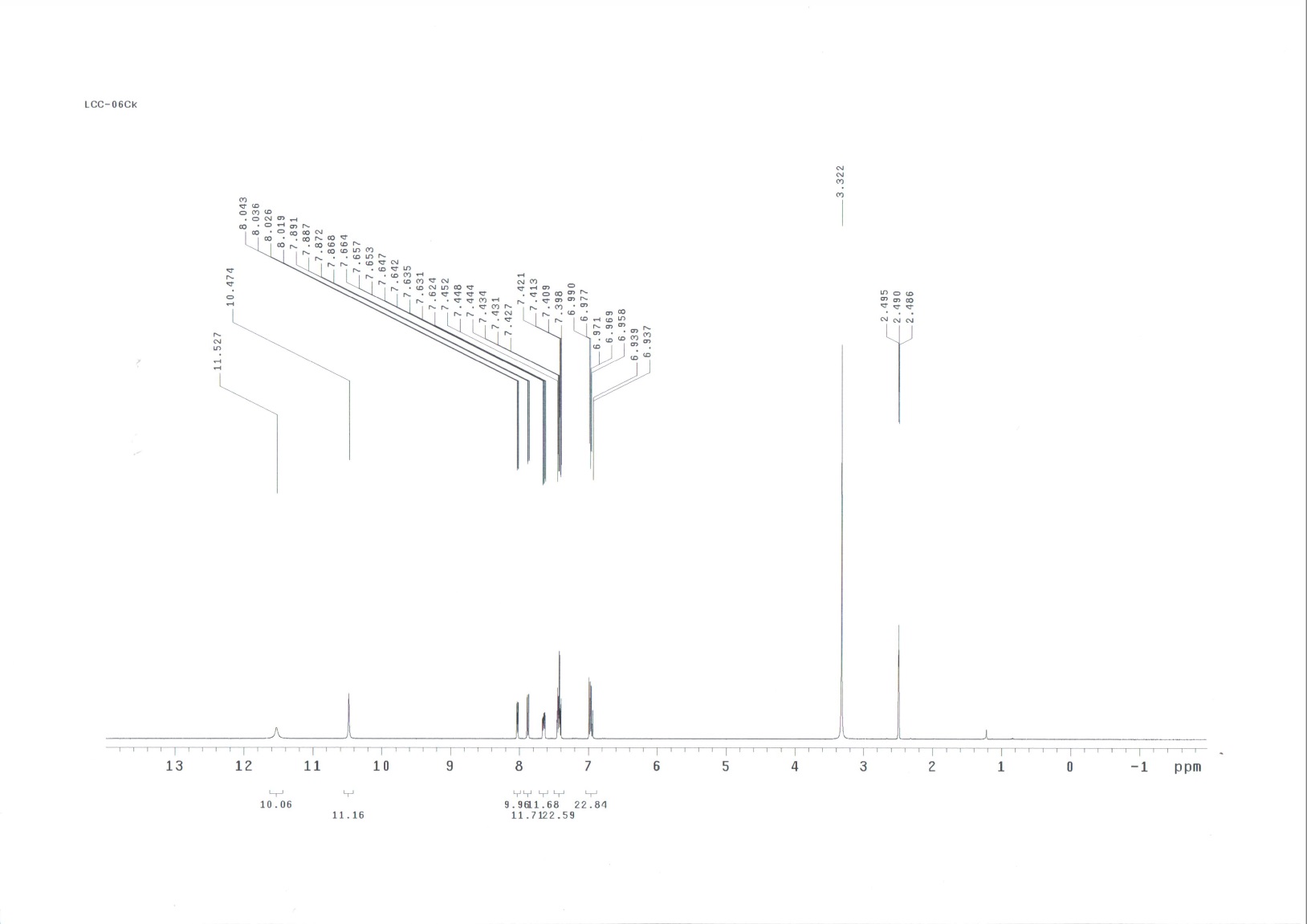
**

(A) ^1^H NMR of HS-Ck (400 MHz, DMSO-*d_6_*)

**
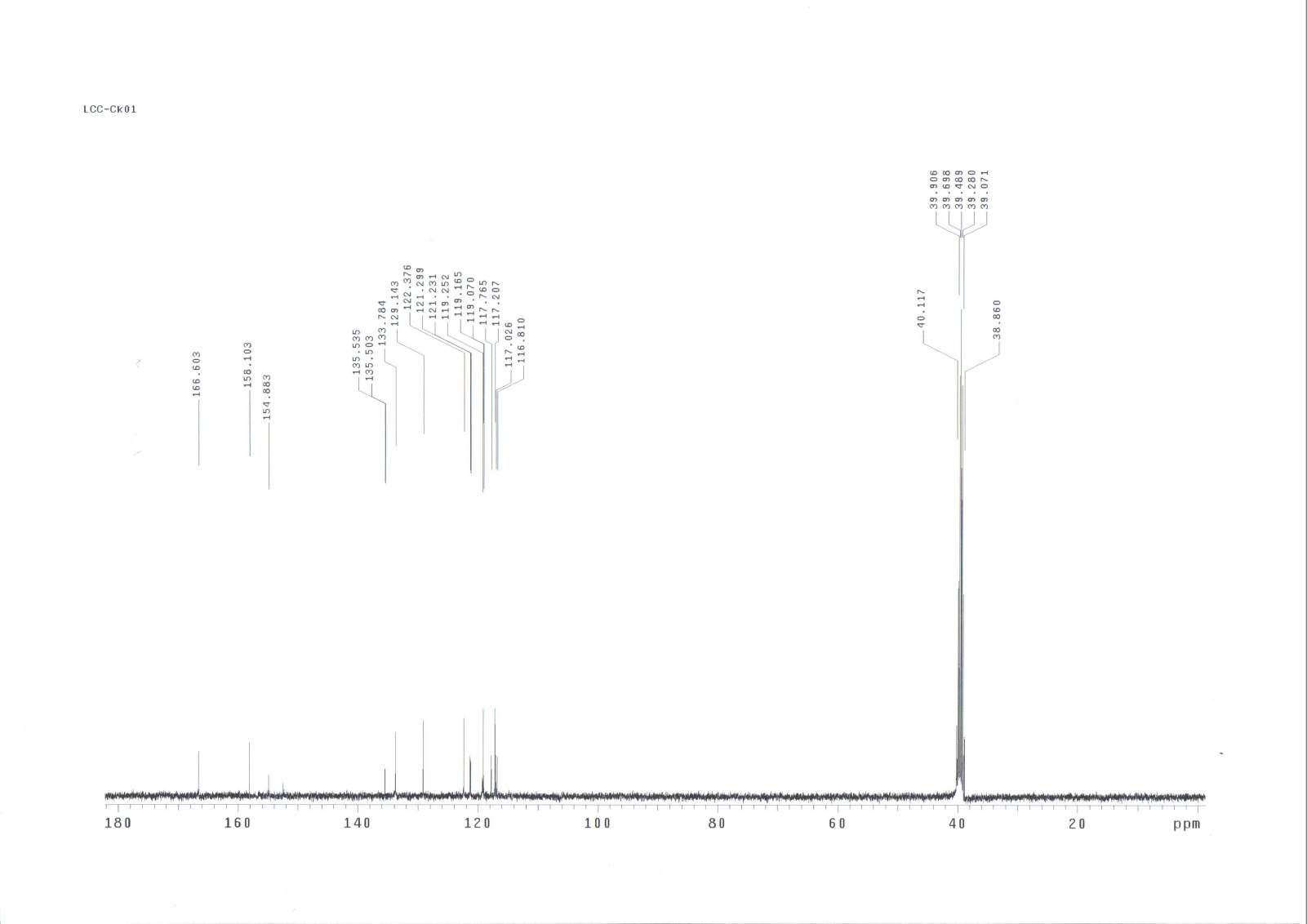
**

(B) ^13^C NMR of HS-Ck (100 MHz, DMSO-*d_6_*)

**
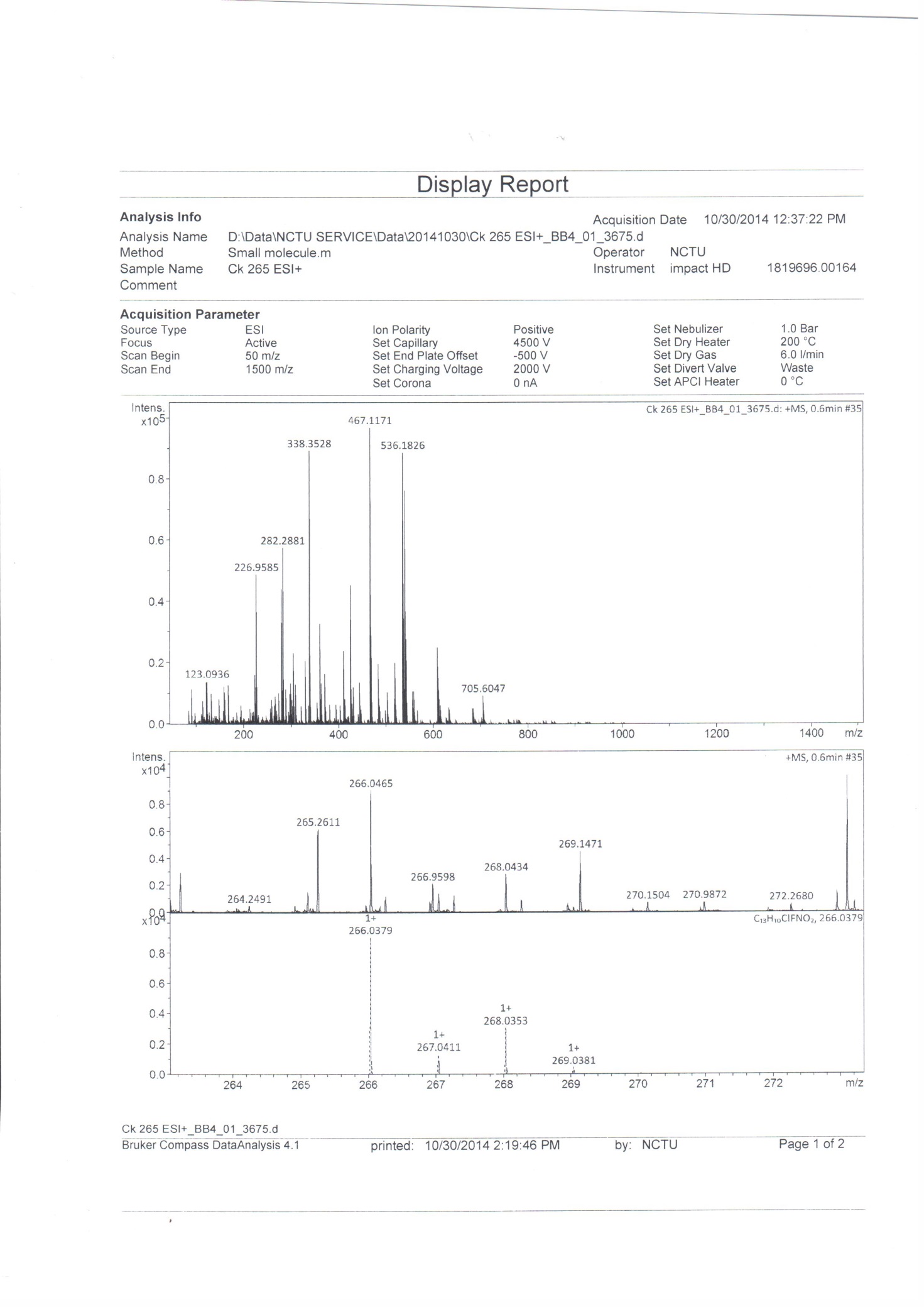
**

(C) HRMS spectra of HS-Ck
